# Local climate and vernalization sensitivity predict the latitudinal patterns of flowering onset in the crop wild relative *Linum bienne* Mill.

**DOI:** 10.1093/aob/mcae040

**Published:** 2024-03-14

**Authors:** Beatrice Landoni, Pilar Suárez-Montes, Rico H F Habeahan, Adrian C Brennan, Rocío Pérez-Barrales

**Affiliations:** School of Biological Sciences, University of Portsmouth, Portsmouth, UK; Department of Biosciences, University of Milan, Milan, Italy; Department of Biosciences, Durham University, Durham, UK; Department of Biosciences, Durham University, Durham, UK; Department of Biosciences, Durham University, Durham, UK; School of Biological Sciences, University of Portsmouth, Portsmouth, UK; Botany Department, University of Granada, Granada, Spain

**Keywords:** Climate change, crop wild relative, flax, flowering phenology, latitudinal gradients, local adaptation, genetic differentiation, vernalization

## Abstract

**Background and Aims:**

The timing of flowering onset is often correlated with latitude, indicative of climatic gradients. Flowering onset in temperate species commonly requires exposure to cold temperatures, known as vernalization. Hence, population differentiation of flowering onset with latitude might reflect adaptation to the local climatic conditions experienced by populations.

**Methods:**

Within its western range, seeds from *Linum bienne* populations (the wild relative of cultivated *Linum usitatissimum*) were used to describe the latitudinal differentiation of flowering onset to determine its association with the local climate of the population. A vernalization experiment including different crop cultivars was used to determine how vernalization accelerates flowering onset, in addition to the vernalization sensitivity response among populations and cultivars. Additionally, genetic differentiation of *L. bienne* populations along the latitudinal range was scrutinized using microsatellite markers.

**Key Results:**

Flowering onset varied with latitude of origin, with southern populations flowering earlier than their northern counterparts. Vernalization reduced the number of days to flowering onset, but vernalization sensitivity was greater in northern populations compared with southern ones. Conversely, vernalization delayed flowering onset in the crop, exhibiting less variation in sensitivity. In *L. bienne*, both flowering onset and vernalization sensitivity were better predicted by the local climate of the population than by latitude itself. Microsatellite data unveiled genetic differentiation of populations, forming two groups geographically partitioned along latitude.

**Conclusions:**

The consistent finding of latitudinal variation across experiments suggests that both flowering onset and vernalization sensitivity in *L. bienne* populations are under genetic regulation and might depend on climatic cues at the place of origin. The association with climatic gradients along latitude suggests that the climate experienced locally drives population differentiation of the flowering onset and vernalization sensitivity patterns. The genetic population structure suggests that past population history could have influenced the flowering initiation patterns detected, which deserves further work.

## INTRODUCTION

The term ‘flowering onset’ refers to the duration between seedling germination or emergence and the initial unfolding of the first flower. In temperate species with broad geographical distributions, this onset varies with latitude (Boudry *et al.*, 2002; Olsson and Ågren, 2002; Stinchcombe *et al.*, 2004; Debieu *et al.*, 2013; Richardson *et al.*, 2016). Such latitude-based variation encapsulates environmental gradients, encompassing factors such as photoperiod, temperature and precipitation, all of which influence the flowering phenology and various life-history traits (Keller *et al.*, 2009; Pau *et al.*, 2011; Colautti and Barrett, 2013; de Frenne *et al.*, 2013; Preite *et al.*, 2015; Burgarella *et al.*, 2016; Muir and Angert, 2017). At finer spatial scales, variation in flowering onset mirrors elevational gradients or topographical heterogeneity, both of which can be correlated with variation in the local climate (Franks *et al.*, 2007; Méndez-Vigo *et al.*, 2011; Halbritter *et al.*, 2018; Lampei *et al.*, 2019; Morente-López *et al.*, 2020). Given the influence of flowering onset on subsequent reproductive events and plant fitness, spatial differences in this onset are commonly interpreted as evidence of an adaptive response to environmental and climatic gradients at multiple geographical scales (Endler, 1977; Boudry *et al.*, 2002; Stinchcombe *et al.*, 2004; Méndez-Vigo *et al.*, 2011; Burgarella *et al.*, 2016; Morente-López *et al.*, 2020). Furthermore, empirical evidence from experimental manipulations and reciprocal transplant experiments have provided further support to the notion that the clinal variation in flowering onset results from adaptation to local environmental and climatic conditions (Ågren and Schemske, 2012; Colautti and Barrett, 2013; Lowry *et al.*, 2019). However, the variation in flowering onset can also be influenced by correlations with life-history traits (Colautti *et al.*, 2010; Haselhorst *et al.*, 2011; Ehrlén, 2015; Auge *et al.*, 2019) or by neutral processes (Leimu and Fischer, 2008; Keller *et al.*, 2009; Chen *et al.*, 2012).

The shift from vegetative growth to the onset of flowering is governed intricately by a complex network of genes that respond to both endogenous cues and exogenous environmental stimuli (Amasino and Michaels, 2010). In temperate species, key pathways within the flowering gene network involve vernalization and photoperiod because temporal trends in temperature and daylength serve as reliable cues for discerning the transitions between seasons. By doing so, plants effectively synchronize their reproduction with the optimal local growing conditions and maximize fitness across the spectrum of environmental conditions within a species range (Amasino and Michaels, 2010; Salomé *et al.*, 2011; Andrés and Coupland, 2012; Blackman, 2017; Bouché *et al.*, 2017; Whittaker and Dean, 2017; Zan and Carlborg, 2019; Friedman, 2020). The synchronization between reproduction and local environmental conditions is evidenced further by the spatial distribution of allelic variation of flowering genes involved in the vernalization and photoperiod pathways along environmental clines (Stinchcombe *et al.*, 2004, 2005; Samis *et al.*, 2008; Keller *et al.*, 2011; Méndez-Vigo *et al.*, 2011; Burgarella *et al.*, 2016), and by the shifts in flowering onset associated with changes in temperature and photoperiod regimes observed in multiple species (Wadgymar *et al.*, 2018). The correlation between allelic variation of flowering genes and environmental gradients can create constitutive differences among populations in the onset of flowering or in the magnitude of the population response to environmental stimuli, in turn depicting clinal variations along latitudinal and elevational gradients (Stinchcombe *et al.*, 2005; Méndez-Vigo *et al.*, 2011; Lewandowska-Sabat *et al.*, 2012; Toftegaard *et al.*, 2016; Prevéy *et al.*, 2017; Thibault *et al.*, 2020). For instance, the responses of flowering onset to vernalization in *Arabidopsis thaliana* have been associated with adaptive responses to different climates, leading to ecotypes displaying differences in flowering time and other life-cycle events across its range (Whittaker and Dean, 2017; Exposito-Alonso, 2020), a pattern also described in other herbaceous species (Boudry *et al.*, 2002; Quilot-Turion *et al.*, 2013). In sea beet, a latitudinal cline in the requirement of vernalization for flowering is correlated with temperature and environmental disturbance, resulting in fast-cycling annual and perennial life histories across its native range (Boudry *et al.*, 2002; Hautekèete *et al.*, 2002).

With the current increase in temperatures and warmer winters attributed to global warming, plant populations now experience different vernalization and photoperiod conditions in their native range. Consequently, many plants are responding by shifting the timing of flowering and other correlated traits, with potential ecological consequences for plant populations (Cook *et al.*, 2012; van Dijk and Hautekèete, 2014). Both standing genetic variation and phenotypic plasticity seem pivotal in facilitating rapid flowering onset responses to abrupt changes in climate in wild plant species (Franks *et al.*, 2007; van Dijk, 2009; van Dijk and Hautekèete, 2014). However, the transition to the onset of flowering is not solely contingent on the environmental conditions preceding flowering; it is also influenced by the developmental stage of the plant and interdependencies between traits (Boudry *et al.*, 2002; Stinchcombe *et al.*, 2004, 2005; Quilot-Turion *et al.*, 2013; Rubin and Friedman, 2018; Gremer *et al.*, 2020; Thibault *et al.*, 2020). For instance, an earlier onset of flowering and a reduction of the plant size threshold for flowering are considered adaptive responses to an increase in aridity or unpredictable rainfall in annual wild species (Reynolds, 1984*a*, *b*; Aronson *et al.*, 1992; Cui *et al.*, 2017). However, in the context of annual crop systems, such responses carry a reduction in yield. In the case of winter annual crops, warmer winters could delay flowering by extending the floral bud dormancy, leading to associated reductions in yield (Lu *et al.*, 2022). The variation in flowering onset and chilling requirements has played a pivotal role in the domestication and adaptation of numerous crops to climates and environmental conditions distinct to their centre of origin (Saisho *et al.*, 2001; Abbo *et al.*, 2002; Casao *et al.*, 2011; Adhikari *et al.*, 2012; Höft *et al.*, 2018). Hence, it is imperative to bridge gaps in our understanding of the environmental factors that predict flowering onset in wild species, especially crop relatives, to predict the responses of species to changing environments (Valladares *et al.*, 2014; Ehrlén and Valdés, 2020), but also to identify genetic variation to enhance crop resilience (Viruel *et al.*, 2021).

In this study, we investigate the variation of flowering onset and its potential causes in *Linum bienne* Mill., the underexplored wild progenitor of cultivated *Linum usitatissimum* L. (Fu, 2023). *Linum bienne* covers a wide geographical range spanning the entire Mediterranean Basin and Western Europe (Fu, 2023). Within this region, the species experiences diverse climatic conditions, from Mediterranean climate, with hot and dry summers and mild winters, to wet oceanic Atlantic climate, featuring cool to warm summers and cool winters. Natural phenotypic variation of *L. bienne* has been described from Turkish populations, revealing that geographical distance and elevation are correlated with variation in a suite of phenotypic traits, including flowering onset. Other studies, including wider germplasm collections, have identified genetic differentiation across the species range (Uysal *et al.*, 2010, 2012; Soto-Cerda *et al.*, 2014). The cultivated *L. usitatissimum* is a crop grown across temperate regions of the world (Zohary and Hopf, 2000; Weiss and Zohary, 2011; Stavropoulos *et al.*, 2023). Globally, within different genetic clusters of the crop, there are associations between genomic regions and latitude, daylength, or mean daily temperature (Sertse *et al.*, 2019). The crop shows variation in its flowering time response to vernalization and photoperiod in varieties adapted to grow at different latitudes (Fu, 2012; Darapuneni *et al.*, 2014). Interestingly, after its domestication in the Near East, the spread of the crop towards northern latitudes in Europe coincided with a change in flowering time and plant architecture, a process likely to be mediated by capturing alleles of flowering time genes from wild local *L. bienne* populations (Gutaker *et al.*, 2019). However, despite the apparent role of *L. bienne* for the initial natural adaptation of the flowering time of the crop from Mediterranean to temperate climate in Europe, the variation in flowering onset across its wider latitudinal range and associated environmental correlates remains unknown.

The overarching goal of this study was to investigate whether the variation in the flowering onset of *L. bienne* populations is associated with gradients in latitude and climate and to explore the potential role of vernalization in the emergence of latitudinal gradients. We hypothesized that flowering onset and its response to vernalization covary with latitude, reflecting an adaptation to the local climate that populations experience. To test this hypothesis, we sampled seeds from *L. bienne* populations within the western part of the species range in Europe to describe population differentiation in flowering onset in controlled conditions in a greenhouse. We also determined whether populations provide different responses in flowering onset when subjected to vernalization. These two experiments served as the basis for evaluating whether latitude of origin and local climate (i.e. the local climate that the *L. bienne* surveyed populations experienced over 30 years) predict flowering onset and vernalization sensitivity among populations. Moreover, to gain better insights into the breadth of variation of flowering onset, we repeated the experiment using a collection of *L. usitatissimum* cultivars to quantify the magnitude of the vernalization sensitivity and compared it with that observed in the surveyed *L. bienne* populations. Finally, using microsatellite markers, we genotyped six populations representing the latitudinal range to discern population genetic structure and differentiation.

## MATERIALS AND METHODS

### Study species and population collection

**Fig. 1.**
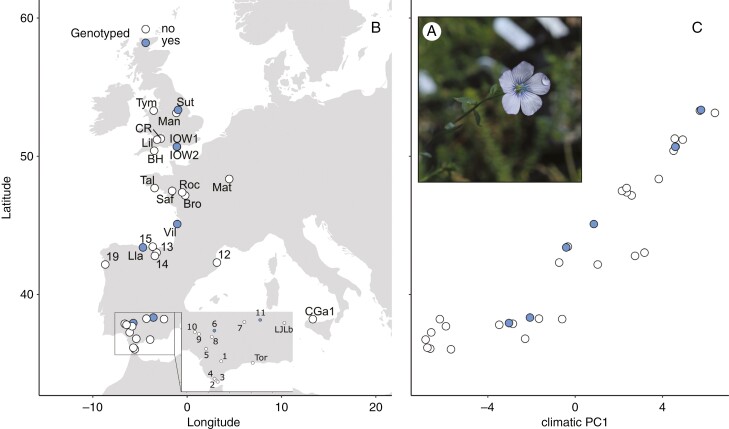
*Linum bienne* (A), the populations surveyed across its western distribution range (B), and the relationship between climatic PC1 and the latitude of origin of the populations surveyed (C). In B and C, circles represent the populations surveyed; blue circles indicate genotyped populations, and white circles the rest of the population. Photograph of *Linum bienne* flower taken by B. Landoni.

*Linum bienne* Mill. is an annual, biennial or perennial herb that boasts a broad geographical distribution encompassing the entire Mediterranean Region, Atlantic Europe and the British Isles, but has also been introduced in California, Chile, Australia and New Zealand (Fu, 2023). It is characteristic of dry and calcareous soils, growing from sea level up to 1200 m. The plants feature slender stems and produce a cymose inflorescence with pale blue flowers. The flowers are homostylous, self-compatible and undergo autonomous self-pollination (upon flower opening, stigmas are receptive and dehisced anthers contact stigmas, B. Landoni and R. Pérez-Barrales, personal observation; Fig. 1A). The fruits are small capsules bearing up to ten seeds (Martínez-Labarga and Muñoz-Garmendia, 2015).

In this study, online databases (Anthos, http://www.anthos.es/; GBIF, https://www.gbif.org/; and BSBI, https://database.bsbi.org/) were used to plan the population survey in the western latitudinal range of the species distribution (Sicily, Spain, France and England). A comprehensive survey involving 34 populations (comprising 851 individual plants per population; Fig. 1B; for specific geographical coordinates and elevation details, see Supplementary Data Table S1) was conducted between 2013 and 2017, with most populations collected in 2016. Field samples were obtained by collecting fruits from a single individual plant (hereafter family) per patch, ensuring ≥1 m distance between patches to prevent sampling the same maternal individual. All collected families, with fruit capsules close to ripening at the time of collection, were stored individually in paper envelopes in a cold room at 4 °C with silica gel to absorb excess humidity. Seeds for one population (Dor) were sourced from Emorsgate Seeds, King’s Lynn, UK (https://wildseed.co.uk). In addition, seeds from 16 cultivated varieties originating from across western Europe and Canada were provided from Flaxland (Stroud, UK), Terre de Lin (Saint-Pierre-le-Viger, France), and the Leibniz Institute of Plant Genetics and Crop Plant Research (Gatersleben, Germany). Details of all cultivated flax samples are included in the Supplementary Data (Table S1).

### Description of historical climate of the populations surveyed

For all *L. bienne* populations sampled in the field, data on mean monthly averages over a 30-year period (1970–2000) for precipitation (in millimetres), solar radiation (in kilojoules per day per square metre), average temperature (in degrees Celsius), minimum and maximum temperature (in degrees Celsius), vapour pressure (in kilopascals) and wind speed (in metres per second) were retrieved using the WorldClim database at 30 arcsec resolution (Fick and Hijmans, 2017). The climatic data were averaged by season. Months were assigned to seasons as follows: winter, December–February; spring, March–May; summer, June–August; and autumn, September–November. A principal component analysis (PCA) was used to reduce the dimensionality of the scaled climatic data. The first three principal coordinates (PCs) were retained because they explained most of the variation observed for each season. To assess the association with latitude, Pearson’s correlation coefficient was used to quantify the extent to which the first three principal components, denoted as climatic PC1, PC2 and PC3 hereafter, were correlated.

### *Characterization of flowering onset of* L. bienne *populations in greenhouse conditions*

The experiment took place in the insect-free greenhouse facility at the University of Portsmouth. An average of 18 families from 32 populations were sown over a week in October 2017 (Supplementary Data Tables S1 and S2). Before sowing, seeds underwent a 48 h imbibition in 5 % gibberellic acid to synchronize germination time. Sowing was randomized across populations and days such that the same set of populations was sown each day. Five seeds from the same family were placed in pots (9 cm × 9 cm × 10 cm) with a 3:2 mix of compost and perlite (the number of seedlings per pot did not affect the results of the analyses; results not shown). The position of populations and families was randomized across the greenhouse. Plants were grown using LED lights (14 h light at 22 °C and 10 h dark at 16 °C) throughout the experiment. Regular irrigation using an ebb-and-flow system occurred three times a week, supplemented by the application of commercial fertilizer every two months through plant spraying. Given that *L. bienne* is susceptible to powdery mildew (*Oidium* spp.), pest control was done using a sulphur burner. From mid-June to early September, plants were moved outside the greenhouse to prevent heat stress owing to the absence of an automatic cooling system, returning to the facility in September. During this period, plants were watered three times a week. The experiment concluded in October 2018.

Seedling emergence, defined as observation of two cotyledons, was monitored twice a week for two months. Adult plants were inspected three days each week to record flowering onset, measured as the number of days between the sowing date and the first flowering for each family. During the period when plants remained outside the greenhouse facility, insect visitation was minimal (R. Pérez-Barrales, personal observation). Considering that autonomous self-pollination happens upon flower opening, with negligible likelihood of outcross pollination, seed production was assumed to be derived primarily from self-pollination (cultivated flax has been estimated to have >95 % selfing rate, with gene flow <2 % over 10 cm distance in the field; Jhala *et al.*, 2011). Fruits were collected into separate paper envelopes per family as they ripened (*F*_1_ generation) and used in the subsequent vernalization experiment.

Pearson’s correlation coefficient was used to explore the relationship between flowering onset data acquired in the greenhouse and the latitude of origin of populations, climatic PC1, PC2 and PC3. Using linear regression models, we characterized the slope between the population mean of flowering onset generated in the greenhouse and the latitude of origin and climatic PC1. Model selection was applied to determine whether the latitude of origin or climatic PC1 better predicted average population flowering onset. This involved comparing all combinations of latitude and climatic PC1, encompassing a single predictor, an additive effect model, and an additive effect model incorporating the interaction term PC1 × latitude. The comparisons were made using the change in the Akaike information criterion corrected for small sample size (ΔAICc) in the AICcmodavg R package v.1.25 (Mazerolle, 2012). A general linear model with binomial distribution and probit link function was then used to describe the relationship between the proportion of families that flowered in each population with latitude of origin.

### Flowering onset in response to vernalization

The *F*_1_ generation of 28 *L. bienne* populations from the preceding experiment and 16 *L. usitatissimum* cultivars were used to quantify the sensitivity of flowering onset (i.e. number of days to advance or delay) to vernalization (Supplementary Data Tables S1 and S2). The selected *L. bienne* populations effectively represented the latitudinal range covered in the study. In both species, up to five seeds for each family or cultivar were sown in pots (10 cm × 9.5 cm × 10 cm) filled with a 3:1 mix of compost and perlite and grown in controlled environment chambers (models A3655 and A3658, Weiss-Gallenkamp, Loughborough, UK). Families within populations (for *L. bienne*) or cultivars (for *L. usitatissimum*) were arranged in a randomized block design, including two replicates for each family or cultivar, and with each treatment replicated across two growth chambers. The experimental conditions mirrored those established for *L. bienne* in previous research (Gutaker, 2014). Seeds underwent a 3-day cold stratification period in darkness at 4 °C, followed by 10 days at 22–20 °C for 16 h–8 h in light–dark photoperiod to synchronize the germination. Then, for the next 40 days, half the plants were exposed to vernalization conditions (4 °C, 16 h–8 h light–dark photoperiod), while the rest were maintained in standard (no vernalization) conditions (24–16 °C and 16 h–8 h light–dark photoperiod). After completing the vernalization treatment, plants from both treatments were subjected to the same conditions (24–16 °C, 16 h–8 h light–dark photoperiod). Seedling emergence was recorded twice a week for two weeks; survival was monitored until blooming (excluding plants that perished before blooming), and plant size was quantified as the number of basal branches when the first flower opened. Flowering onset, measured as the number of days from sowing until the first flower opened in each pot, was recorded five times a week until the experiment was concluded after 320 days.

After accounting for seedling emergence and plant mortality, the collected data for *L. bienne* included one to seven families and 2–12 plants per population and treatment. For *L. usitatissimum*, the data were collected for two to four plants per cultivar and treatment (Supplementary Data Table S2). Linear mixed-effect models were used to analyse flowering onset responses to vernalization across populations of *L. bienne* and cultivars of *L. usitatissimum*. The model for *L. bienne* incorporated the fixed-effect terms treatment, population, and plant size, along with the interaction term treatment × population. Random factors consisted of family nested within population, and block nested within treatment and growth chamber (treatment: growth chamber: block). The model for *L. usitatissimum* included the fixed-effect terms treatment, cultivar, and plant size, and the interaction terms treatment × cultivar. The random factor was block nested within treatment and growth chamber (treatment: growth chamber: block). Linear mixed-effect models were computed with the R package lme4 v.1.1-7 (Bates *et al.*, 2015). The statistical significance of the fixed-effect terms was assessed with the ANOVA function (type II sum of squares) as implemented in the R package car (Fox *et al.*, 2012). Models were summarized with the package lmertest, based on Satterthwaite’s methods (lmertest, Kuznetsova *et al.*, 2017; RLRsim, Scheipl *et al.*, 2008).

Vernalization sensitivity (VRNs) was used to quantify the extent of the flowering onset response to the vernalization treatment. Following Falconer (1990), vernalization sensitivity calculated the difference between the measurement of a population or cultivar in different environments, thereby serving as a metric for the plastic response of the genotype:


$$\begin{array}{l} VRNs=\frac{\left({\bar{X}}_{pop}^{no-vrn}-{\bar{X}}_{pop}^{vrn}\right)}{\left({\bar{X}}^{no-vrn}-{\bar{X}}^{vrn}\right)} \end{array}$$

Here, ${\bar{X}}_{pop}^{no\text{-}vrn}$ and ${\bar{X}}_{pop}^{vrn}$ represent the population (or cultivar) mean values of flowering onset for the no vernalization and vernalization treatment, respectively, while ${\bar{X}}^{no-vrn}$ and ${\bar{X}}^{vrn}$ represent the grand mean value of the no vernalization and vernalization treatment, respectively. In the case of *L. bienne*, for which the vernalization treatment typically resulted in a reduction of the number of days to flowering onset (see Results), positive values represent earlier flowering onset, whereas negative values indicate later flowering of vernalized plants (i.e. a reduction or increase of days to flowering, respectively). Conversely, the general vernalization response for the *L. usitatissimum* cultivars used here was opposite to that in *L. bienne*, such that positive sensitivity values indicate later flowering onset after vernalization. In both species, values close to zero indicate less sensitivity to vernalization. For *L. bienne* populations only, model selection was then used to assess whether vernalization sensitivity could be predicted better by latitude of origin or by PC1, as described above. Additionally, linear models were applied to describe the slope of the relationship of vernalization sensitivity to latitude of origin and PC1. To gain a better understanding of the sensitivity response between *L. bienne* and *L. usitatissiumum*, the absolute value of vernalization sensitivity, indicative of the response magnitude, was compared in two ways. With the R package bmbstats (Jovanovic, 2020), bootstrapped confidence intervals were calculated for: (1) the difference in absolute vernalization sensitivity mean values between the two species, and (2) the difference in absolute vernalization sensitivity standard deviation (s.d.) between the two species.

### Flowering onset comparisons across experiments

Pearson’s correlation coefficient was used to investigate the robustness of flowering onset responses obtained in the different generations (*F*_0_ and *F*_1_) and experimental conditions (no vernalization and vernalization) to determine the fixed genetic influence of population of origin.

### Population genotyping and population genetic structure

To investigate whether the observed phenological patterns might be influenced by underlying population genetic structure, we conducted genotyping on families from six populations representing the southern and northern latitudinal edges of the species (Fig. 1B; Supplementary Data Table S1). Genotyping was conducted following Landoni *et al.* (2020), using microsatellite markers developed *ad hoc* for population genetic studies in *L. bienne*. Of the 16 microsatellite markers developed, 15 exhibited signs of duplication, manifesting as high heterozygosity. Consequently, each allele at each locus was treated as a dominant (present or absent) locus, resulting in a total of 64 dominant loci. Three of these dominant loci were excluded from further analyses because a unique allele was fixed across populations.

The genetic structure of the six populations was explored using Bayesian clustering with the software STRUCTURE v.2.3.4 and the StrAuto pipeline (Pritchard *et al.*, 2000; Chhatre and Emerson, 2017). For Bayesian clustering, we used the admixture model following Hardy–Weinberg equilibrium and correlated allele frequencies. Run parameters included ten independent replications with a burn-in period of 50 000, followed by 1 000 000 Markov chain Monte Carlo iterations, with the number of genetic clusters (*K*) varying from one to six. To determine the optimal number of clusters, we calculated the *ad hoc* measure Δ*K*, according to Evanno *et al.* (2005). The same STRUCTURE analysis was repeated treating the markers as co-dominant. After running STRUCTURE on the dominant and co-dominant datasets, runs for the optimal value of *K* were compared using Clumpak (Kopelman *et al.*, 2015). The comparison allowed us to assess whether, for the same value of *K*, specifying markers as dominant or co-dominant resulted in similar assignment of individuals to clusters. In addition, discriminant analysis of principal components (DAPC) was performed with the R package adegenet v.2.1.3 (Jombart *et al.*, 2010). For the DAPC analysis, the optimal number of clusters and individual probabilities of assignment to clusters was identified using find.clusters() following dapc() using the R package adegenet v.2.1.3. The genetic clustering results were plotted with the aid of the R package pophelper v.2.3.0 (Francis, 2016).

All statistical analyses in the present study were executed in R (R Core Team, 2018; see detailed description of R packages above). All plots were produced with the R package ggplot2 v.3.3.3 (Wickham, 2016) together with Inkscape v.1.1.1 (Inkscape Project, 2020).

## RESULTS

### Description of historical climate experienced by the populations surveyed

The first principal component (PC1) accounted for 64.9 % of the variation in the local climate of the sampled *L. bienne* populations. The variables that contributed more to PC1 included the average and maximum temperature in autumn and spring, maximum temperature in winter, minimum temperature in summer (temperature variables made similar contributions) and, to a lower extent, solar radiation of all seasons and summer precipitation. The second principal component (PC2) explained 18.6 % of the variation, with wind speed and vapour pressure, winter minimum temperature and precipitation during autumn and winter being the variables making the most contribution. The third principal component (PC3) explained 10 % of the variance, and the variables that explained most of the variation along its axis were autumn, winter and spring precipitation. Supplementary Data Fig. S1A, B includes plots of PC1 against PC2 and PC3, respectively, and Supplementary Data Fig. S1C includes the contribution of the climatic variables to PC1, PC2, and PC3. The correlation between population PC1 scores and latitude of origin was statistically significant and positive (Fig. 1C; Supplementary Data Table S3). In contrast, the relationship between latitude of origin and PC2 and PC3 was not statistically significant (Supplementary Data Table S4).

### *Characterization of flowering onset of* L. bienne *populations in greenhouse conditions*

Seedlings from 784 of 854 families emerged within one month, averaging three plants per pot. Mortality before flowering occurred in ~0.08 % of seedlings, resulting in all 784 emerged families being represented by at least one seedling when plants started to flower. Out of the total emerged families, 444 families flowered during the 1-year experiment. The remaining families were still alive by the time the experiment concluded. The earliest population to flower was population 9 (mean = 129 days), and the latest was population BH (mean = 228 days), with flowering, on average, commencing 190 days after sowing (Supplementary Data Table S2). Correlations with flowering onset were statistically significant for latitude of origin (*r* = 0.70, *P* < 0.0001) and climatic PC1 (*r* = −0.78, *P* < 0.0001), but not for PC2 (*r* = 0.06, *P* > 0.05) or PC3 (*r* = −0.02, *P* > 0.05; Supplementary Data Table S4). Hence, PC2 and PC3 were excluded from further analyses.

Using model selection based on ΔAICc, it was found that the model explaining most of the variation in flowering onset (60 % based on adjusted *r*^2^) included only the climatic PC1, in contrast to models with only latitude of origin or with both latitude of origin and climatic PC1 (Table 1). The linear regression model revealed that flowering onset was delayed by ~3 days per degree of latitude going north (Table 1). The relationship between flowering onset and PC1 was positive, indicating that flowering onset was delayed by ~5 days for every increased unit of climatic PC1 (Table 1). The relationship between the proportion of flowering families per population and latitude of origin was negative (intercept = 7.79 ± 0.65, slope = −0.17 ± 0.01, χ^2^ = 162.1, d.f. = 1, *P* < 0.001), suggesting that, at the population level, fewer families from northern latitudes flowered within the duration of the experiment.

**Table 1.** Model selection to predict flowering onset (greenhouse data) and vernalization sensitivity according to latitude and local climate among *Linum bienne* populations. The results of model selection are included for the models tested (univariate models with only latitude or PC1, additive model, a model with the interaction term, and the null model with only the intercept), including the number of terms of the model (*K*), AICc values, the difference with regard to the model with the lowest value (ΔAICc), and the parsimony weighting of support comparing these models (Weights AICcWt). Also shown are the summary results, including: the intercepts, slopes, *P*-values, residual degrees of freedom (d.f.res), *r*^2^ and adjusted *r*^2^ of the univariate linear models between flowering onset and vernalization sensitivity with latitude and PC1.

Experiment	Response variable	Model selection					Model	Predictor	Estimate	*P*-value	d.f.res	*r* ^2^	Adjusted *r*^2^
		Predictors	*K*	AICc	ΔAICc	Weights AICcWt							
Greenhouse	Flowering onset (days from sowing)	PC1	3	280.2	0	0.61	PC1	Intercept	188.91	<0.0001	30	0.61	0. 60
		PC1 × latitude	5	282.6	2.37	0.19		PC1	5.30	<0.0001			
		PC1 + latitude	4	282.6	2.4	0.18							
		Latitude	3	288	7.79	0.01	Latitude	Intercept	43.03	n.s.	30	0.5	0.48
		Null	2	307.8	27.58	0		Latitude	3.37	<0.0001			
Vernalization	Vernalization sensitivity	PC1	3	52.21	0.00	0.48	PC1	Intercept	1.07	<0.001	20	0.26	0.22
		Latitude	3	53.07	0.85	0.31		PC1	0.09	<0.01			
		PC1 + latitude	4	55.19	2.97	0.11							
		PC1 × latitude	5	57.53	5.32	0.03	Latitude	Intercept	1.70	n.s.	20	0.23	0.19
		Null	2	56.16	3.95	0.07		Latitude	0.06	0.02			

### Flowering onset in response to vernalization

**Fig. 2.**
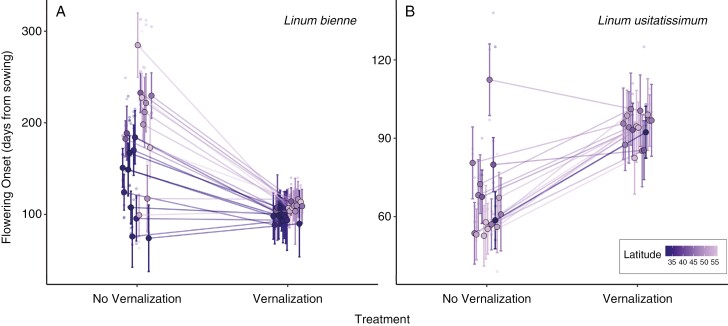
Reaction norm of flowering onset measured since seed sowing in *Linum bienne* populations (A) and in *Linum usitatissimum* cultivars (B) in response to the vernalization manipulation. Points and error bars indicate the mean and s.d. of flowering onset per population (or cultivar) and treatment, and lines link populations (or cultivars) across treatments. The colour scale represents the latitude of origin of the *L. bienne* populations used in the experiment. For *L. usitatissimum*, the latitude of origin is represented by the centroid of the country of origin of the cultivar.

For *L. bienne*, 254 plants flowered during the experiment. Of these, 103 plants from 69 families reached flowering in the no vernalization treatment, and 151 plants from 82 families in the vernalization treatment (Supplementary Data Table S2). Three populations in the no vernalization treatment and one population in the vernalization treatment did not flower at all, and three populations were represented in only one of the treatments (Supplementary Data Table S2). Hence, these seven populations (7, 11, 12, 13, 14, Dor and Sut) were excluded from the mixed-effect model analysis. Table 2 includes the results of the analysis of the flowering onset response after vernalization, and Supplementary Data Table S5 includes the estimated values of the model. On average, flowering onset (in days from sowing) in vernalized plants of *L. bienne* occurred earlier (mean = 104, s.d. = 11, *n* = 134) than in non-vernalized plants (mean = 176, s.d. = 66, *n* = 99; Fig. 2A). The linear mixed-effect model explained 81 % of the variation in flowering onset, with all fixed-effect terms (plant size, population, treatment and population × treatment interaction) being statistically significant (Table 2). The positive relationship between plant size and flowering onset (Supplementary Data Table S5) suggests that plants taking longer to shoot the first flower also had more branches. After accounting for plant size, the statistically significant effect of the term population suggests constitutive differences among populations for the differentiation in flowering onset regardless the environmental conditions (Table 2). The statistically significant and negative effect of the treatment (Supplementary Data Table S5) suggests a general response of a reduction in the number of days to flowering onset after vernalization. Finally, the statistically significant interaction term population × treatment indicates that populations are differentiated not only for flowering onset, but also for their response to vernalization, resulting in vernalization responses of different magnitude depending on the population (Fig. 2A; Supplementary Data Table S3).

**Table 2.** Linear mixed models to test the effect of vernalization on flowering onset in *Linum bienne* populations and *Linum usitatissimum* cultivars. The summary results, including the estimates for the intercept and predictor, confidence intervals, *P*-values and degrees of freedom, are reported in Supplementary Data Tables S5 and S6.

	*Linum bienne*				*Linum usitatissimum*		
Predictors	χ^2^	d.f.	*P*-value	Predictors	χ^2^	d.f.	*P*-value
Number of stems	14.27	1	0.0002	Number of stems	37.306	1	<0.0001
Population	111.77	21	<0.0001	Cultivar	57.382	15	<0.0001
Treatment	113.72	1	<0.0001	Treatment	141.728	1	<0.0001
Population:treatment	191.23	21	<0.0001	Cultivar:treatment	42 615	15	0.0002

Vernalization sensitivity varied substantially among *L. bienne* populations (mean = 1.03, s.d. = 0.75, *n* = 22; range: −0.17, 2.65), ranging from large positive values, indicating a considerable advance in flowering onset for many populations after vernalization, to a few populations exhibiting small negative values associated with a flowering onset delay under vernalization (Supplementary Data Table S2). Vernalization sensitivity varied positively with latitude of origin such that towards northern latitudes, populations expressed a stronger reduction in flowering onset (Table 1). Following model selection and ΔAICc, it was found that the model that better predicted vernalization sensitivity included only PC1 (Table 1). The positive relationship between vernalization sensitivity and PC1 suggests that populations displaying greater vernalization sensitivity (i.e. larger advance in flowering onset) were associated with an overall colder climate and wetter summer, whereas populations displaying small vernalization sensitivity (i.e. no advance or small delay in flowering onset) corresponded to warmer climates with drier summers.

For *L. usitatissimum*, all 97 pots sown (51 and 46 exposed to the no vernalization and vernalization conditions, respectively) presented at least one emerged seedling. Mortality was negligible, and 97 pots presented plants that reached flowering (51 in the no vernalization treatment and 46 in the vernalization treatment). Compared with *L. bienne*, *L. usitatissimum* plants flowered earlier, but the vernalization response was opposite. On average, vernalized plants onset flowering at 98 days (s.d. = 7 days, *n* = 46), and no vernalized plants flowered earlier (mean = 70 days, s.d. = 19 days, *n* = 51; Fig. 2B). About 76 % of the variation in flowering onset was explained by the mixed-effect model, with all fixed-effect terms (plant size, cultivar, treatment and the population × treatment interaction) being statistically significant (Table 2). Like *L. bienne*, plant size had a positive relationship with flowering onset (Supplementary Data Table S6), such that plants with more branches flowered later. After accounting for plant size, differences among cultivars denoted substantial differentiation for flowering onset. Contrarily to *L. bienne*, the treatment effect was negative (Supplementary Data Table S6), and plants exposed to vernalization delayed the onset of flowering. Finally, the statistically significant interaction effect cultivar × treatment indicated that cultivars were differentiated in their flowering onset response to vernalization. Vernalization sensitivity varied substantially among *L. usitatissimum* cultivars (mean = 1.04, s.d. = 0.35, *n* = 16; range: −0.43, 1.75; Supplementary Data Table S2), with relatively large positive values indicative of a delay in flowering onset in all cultivars except for one cultivar exhibiting a negative value corresponding to an advance in flowering onset after vernalization (Fig. 2B).

The analyses using the absolute values of the vernalization sensitivity index showed that the average sensitivity response was similar between species (see values above; mean difference = 0.019; bootstrapped confidence interval = [−0.350, 0.369]). In contrast, *L. bienne* showed a substantially higher variability in the magnitude of vernalization sensitivity (represented by the s.d.) than *L. usitatissimum* (s.d. difference = 0.832, bootstrapped confidence interval = [0.699, 1.069]).

### Flowering onset comparisons across all experiments

The correlation between days to flowering onset of the *F*_0_ and *F*_1_ plants (Supplementary Data Table S4) was statistically significant and ranged between *r* = 0.6 (*F*_0_ data from the greenhouse and *F*_1_ data of the plants in the no vernalization treatment) and *r* = 0.8 (*F*_0_ data from the greenhouse and *F*_1_ data of the plants in the vernalization treatment). The correlation between flowering onset of the *F*_1_ plants (vernalized vs. non-vernalized) was also statistically significant (*r* = 0.6; Supplementary Data Table S4). This shows that the change in flowering onset between *F*_0_ and *F*_1_ was similar across experiments, regardless of generation. The varying magnitude of correlation coefficients might be attributable to variation in growing conditions, maternal effects and/or variation in the collection of observations across experiments.

### Population genetic structure

**Fig. 3.**
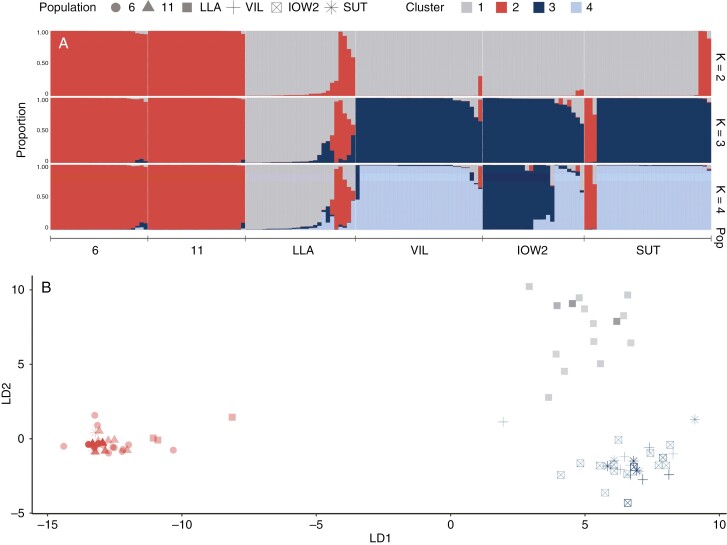
Genetic clustering of six *Linum bienne* populations, where colours indicate clusters obtained with STUCTURE (A) or DAPC (B). (A) STRUCTURE results for two to four genetic clusters indicated with different colours are shown in each row, starting from the top. Sample individuals are ordered along the *x*-axis according to population and the proportion of assignment to the different clusters. Thus, the same individual might appear in a different position along the x-axis for the different Ks. The colours of each vertical bar indicate the mean probabilities of assignment to each genetic cluster. (B) Discriminant analysis of principal components (DAPC) results, showing samples separated according to the primary and secondary discriminant axes. Sample points are coloured according to the optimum number of genetic clusters (*K* = 3), and symbols indicate the populations.

Both Bayesian clustering and k-means clustering, as implemented in DAPC, concurred in identifying the optimal number of genetic clusters as two (Supplementary Data Fig. S2). The first cluster (southern cluster) contained populations 6 and 11 from southern Spain, and the second cluster (northern cluster) contained population LLA from northern Spain, population VIL from France and populations IOW2 and SUT from the UK (*K* = 2). Notably, the Bayesian clustering analysis allocated two individuals in population LLA to the southern cluster and detected a subset of admixed individuals between the southern and northern clusters for *K* = 2 or higher, implying potential gene flow from the southern to the northern cluster. For values of *K* higher than two, the northern cluster exhibited further subdivision into its constitutive populations, with population LLA constituting a standalone cluster (Fig. 3A, B). Only three individuals from population SUT consistently demonstrated assignment to the southern cluster across methods, hinting at possible gene flow, although sample mishandling cannot be excluded, and caution is warranted in drawing definitive conclusions. All markers displayed signs of duplication, prompting a recommendation for their treatment as dominant markers (Landoni *et al.*, 2020). Nevertheless, it is noteworthy that the STRUCTURE analysis, when repeated assuming co-dominance, yielded similar cluster membership for *K* = 2, as shown by the comparison run with Clumpak for dominant and co-dominant STRUCTURE results (Supplementary Data Table S7).

## DISCUSSION

### Clinal variation in flowering onset

Population differentiation in flowering onset along a latitudinal gradient is a pattern frequently attributed to adaptive responses to local environmental conditions and climate, optimizing plant growth and fitness within the growing seasons (Endler, 1977; Boudry *et al.*, 2002; Olsson and Ågren, 2002; Stinchcombe *et al.*, 2004; Keller *et al.*, 2009; Colautti and Barrett, 2013; Preite *et al.*, 2015; Richardson *et al.*, 2016). These clines create selective pressures on phenological traits, fostering genetic differentiation among populations (Méndez-Vigo *et al.*, 2011; Ågren and Schemske, 2012; Burgarella *et al.*, 2016; Lowry *et al.*, 2019). In our study, we quantified flowering onset in controlled greenhouse conditions using *L. bienne* seeds collected from wild populations along a latitudinal range in western Europe, revealing substantial differences in population mean values. In the greenhouse, southern populations initiated flowering earlier than their northern counterparts. Model selection analysis showed that PC1, an axis summarizing climatic conditions experienced by the populations sampled over 30 years, predicted flowering onset better than latitude of origin alone. The findings collectively indicate that the initiation of flowering of *L. bienne* populations across its western latitudinal range is likely to result from the abiotic pressures exerted by the climatic gradient delineated by PC1, in which variables representing temperature, solar radiation and summer precipitation had more weight. The patterns detected align with previous findings that climatic gradients drive selection on phenological traits (Keller *et al.*, 2009; Pau *et al.*, 2011; Colautti and Barrett, 2013; de Frenne *et al.*, 2013; Preite *et al.*, 2015; Burgarella *et al.*, 2016; Muir and Angert, 2017). Our results echo previous observations of population differentiation of various traits along elevational gradients in Turkish populations of *L. bienne*, with flowering onset showing a positive relationship with elevation, hence populations at higher elevation flowered later (Uysal *et al.*, 2012). Our study showed significant associations between flowering onset, latitude of origin and the local climate of populations. However, the decline in the proportion of families that flower within populations with the latitude of origin (i.e. families that did not get to flower by the time the experiment concluded after a year) and the fact that PC1 was predominantly represented by temperature variables suggest that exposure to cold winter temperatures plays a crucial role for the onset of flowering and the observed population differentiation.

### *Sensitivity in the flowering onset response to vernalization in* L. bienne *and its crop*

Vernalization is a mechanism that accelerates the start of phenological events, including flowering. In species covering a wide geographical range, differentiation in vernalization sensitivity is pivotal to trace seasonal environmental changes and to time reproduction to the optimal local climatic conditions (Boudry *et al.*, 2002; Stinchcombe *et al.*, 2004). The validation of vernalization as a mechanism advancing the flowering onset of *L. bienne* revealed patterns aligning with those found in species sharing similar geographical distributions (Boudry *et al.*, 2002; Burgarella *et al.*, 2016; Exposito-Alonso, 2020). The population × treatment effect underscored significant among-population variation in the magnitude of the vernalization response, in line with a substantial population genetic differentiation for that trait. This lends support to the hypothesis that population differentiation in flowering onset might be determined by a response to temperature cues (Boudry *et al.*, 2002; Stinchcombe *et al.*, 2005; Méndez-Vigo *et al.*, 2011; Lewandowska-Sabat *et al.*, 2012; Quilot-Turion *et al.*, 2013). The variation in vernalization sensitivity points to the importance of autumn and winter cold temperatures, particularly for plants from northerly latitudes (van Dijk and Hautekèete, 2014; Blackman, 2017; Prevéy *et al.*, 2017). This was supported by the association of vernalization sensitivity with the climatic gradient represented by PC1 and latitude of origin, wherein populations from the southern range exhibited lower vernalization sensitivity compared with their northern counterparts. Constitutive early flowering and the lack of response to cold cues is often interpreted as an adaptive strategy to escape environmental stress during reproduction, for example in environments characterized by seasonal droughts (Boudry *et al.*, 2002; Exposito-Alonso, 2020). Conversely, higher vernalization sensitivity might reflect adaptation to cold temperatures and shorter growing seasons at higher latitudes (Boudry *et al.*, 2002). The hypothesis that vernalization sensitivity might be driven by climatic gradients is supported further by the flowering onset data obtained in the greenhouse, as evidenced by the decline in the number of flowering families per population with latitude of origin (see discussion above). In other species, population differences in flowering onset and vernalization sensitivity generate intraspecific variability in life histories (Friedman, 2020), an understudied aspect of *L. bienne* that deserves attention. Most *L. bienne* families from the selected populations flowered without vernalization, and the positive relationship between plant size and flowering onset suggests that plant size, together with vernalization requirements, is a crucial determinant for the initiation of the reproductive phase in *L. bienne*. Vernalization itself can affect plant size and architecture (Stinchcombe *et al.*, 2005; Méndez-Vigo *et al.*, 2011; Adhikari *et al.*, 2012; Quilot-Turion *et al.*, 2013). The intricate relationship between plant size, vernalization requirements and flowering onset warrants further exploration in future studies in *L. bienne*.

The inclusion of cultivated *L. usitatissimum* sourced from western Europe and Canada has provided a valuable framework for comparative analysis with its wild progenitor, shedding light on the spectrum of vernalization responses influencing the onset of flowering. Overall, *L. usitatissimum* exhibited earlier flowering onset compared with its wild relative, with similar sensitivity values. However, *L. bienne* expressed wider variation in vernalization sensitivity. The general crop response was to delay flowering onset after vernalization, a pattern observed only in some southern populations of *L. bienne*. A noteworthy exception to the crop general behaviour was the northernmost cultivar from Canada, which demonstrated an advance in flowering in response to vernalization, like most *L. bienne* populations. Darapuneni *et al.* (2014) reported similar findings, observing that vernalization delayed flowering in winter varieties cultivated in Texas, whereas spring varieties of the Upper US Midwest and Canada were unaffected. Although the cultivar panel used might not fully represent the entire diversity of *L. usitatissimum*, the consistent delay in flowering in response to vernalization suggests a prevalent pattern among European cultivars that accounted for most of the panel used in the study. Like other crops, vernalization in *L. usitatissimum* probably underwent selection to optimize flowering onset at different latitudes for cultivation (Saisho *et al.*, 2001; Abbo *et al.*, 2002). After its domestication in the Middle East, cultivated flax was adopted progressively in Europe, requiring adaptation to environments with strong seasonality in cold winters and daylength (Gutaker *et al.*, 2019). This adaptation, probably facilitated by secondary introgression of *L. bienne* into the gene pool of cultivated flax, could have introduced novel variation in flowering time genes advantageous in northern latitudes, with a consequent change in the architecture of the plant (Gutaker *et al.*, 2019). However, this gene flow event does not rule out the possibility that domestication bottlenecks might have restricted the range of responses to vernalization currently found in the crop. Further research on the genetic controls of vernalization will help us to understand the adaptive nature of flowering initiation in *L. bienne* with latitude, offering valuable insights for breeding programmes aimed at cultivating flax in diverse environments and climates (Sertse *et al.*, 2019).

### Genetic population differentiation

The population genetic analyses focused on a subset of surveyed populations covering the latitudinal range (Fig. 1B). The results revealed genetic differentiation patterns in two distinct groups: one composed of populations from southern Spain and the other spanning from northern Spain up to northern England (Fig. 3A). This geographical structure of genetic clusters mirrors observations in other plant species across the Mediterranean Basin and northern Europe, where similar genetic structure has been linked to range contraction during the Last Glacial Maximum or other ancient climatic events (Hewitt, 1999; Kadereit *et al.*, 2005). Consequently, it is plausible that neutral demographic processes might also have contributed to the latitudinal cline in flowering onset for *L. bienne* (Keller *et al.*, 2009). Future investigations should incorporate more populations along the geographical range of the species to clarify the interplay between population genetic structure and the variation in flowering onset and associated life-history traits (Exposito-Alonso, 2020). Other studies on *L. bienne* have identified phenotypic and genetic differentiation linked to local adaptation, albeit over smaller geographical scales (Uysal *et al.*, 2010, 2012; Gutaker *et al.*, 2019). The distinctive genetic and phenotypic differences between the southern and northern populations deserves further research. For instance, the distribution of flowering onset variation and its underlying causes should be scrutinized within these genetic groups, particularly in the context of adaptation to local climatic conditions and evolution of life-history strategies (Exposito-Alonso, 2020).

## Conclusions

Our study unveiled a correlation between flowering onset and vernalization sensitivity in *L. bienne* populations along latitude in the west of the native range of the species. With increasing latitude of origin, the onset of flowering was delayed and vernalization sensitivity increased. These patterns were predicted better by the local climate of origin than by latitude *per se*. The cline in flowering onset and its vernalization sensitivity are likely to stem both from population adaptation to the diverse climates experienced, from Mediterranean to an oceanic Atlantic climate, and from genetic differentiation of populations. The consistency of the results among experiments using *F*_0_ and *F*_1_ generations emphasizes the genetic control and genetic variation governing flowering onset among populations. It also shows that maternal effects were probably negligible during the experiments, making our data robust. However, the microsatellite data suggest that the genetic structure of populations, and thus neutral evolutionary events, might also contribute to the geographical distribution of flowering onset and vernalization response. Although our study underscores the significance of the vernalization pathway to time flowering onset, other pathways, such as photoperiod and daylength, could contribute to the variation in flowering and other life-history traits, deserving future research. This knowledge is crucial for predicting phenological responses of the species in the face of a changing climate. Our results not only shed light on the intrinsic variation within *L. bienne* but also hold potential value for the improvement of *L. usitatissiumum*. Altogether, the diverse collection of *L. bienne* populations, coupled with the detailed description of flowering onset and vernalization, enriches our understanding of a poorly studied crop wild relative across a wide latitudinal range.

## SUPPLEMENTARY DATA

Supplementary data are available at *Annals of Botany* online and consist of the following.

Table S1: surveyed *Linum bienne* populations and cultivars of *Linum usitatissimum*, with indication of the type of species (wild for *L. bienne*, type of cultivar as oilseed or fibre for *L. usitatissimum*). Table S2: summary results of the days to flowering onset measured in *Linum bienne* populations in the greenhouse experiment (*F*_0_ generation) and in the vernalization experiment (*F*_1_ generation), and the vernalization sensitivity (Vern. Sensitivity) for *L. bienne* populations and *Linum usitatissimum* cultivars. Table S3: population scores of the PC axes PC1–PC3 retrieved from the principal component analysis using climatic variables obtained from WorldClim. Table S4: Pearson’s correlations between climatic PC1, PC2 and PC3 and flowering onset of *Linum bienne* populations measured in the greenhouse (flowering onset *F*_0_) and in the vernalization experiment using the *F*_1_. Table S5: estimated values of the fixed terms, interaction terms and random factors derived from the linear mixed-effect model on flowering onset of *Linum bienne* in the vernalization experiment. Table S6: estimated values of the fixed terms, interaction terms and random factors derived from the linear mixed-effect model on flowering onset of *Linum usitatissimum* in the vernalization experiment. Table S7: results including the mean proportional membership of individuals across STRUCTURE runs obtained with Clumpak (option ‘Compare’) for *K* = 2, when STRUCTURE was run considering microsatellite markers as dominant or co-dominant. Figure S1: results of the principal component analyses to summarize the local climate of the populations surveyed along the latitudinal gradient. Figure S2: Evanno’s method plot to retrieve optimal number of clusters for *Linum bienne* based on STRUCTURE output.

mcae040_suppl_Supplementary_Tables

mcae040_suppl_Supplementary_Figures

## Data Availability

The data and annotated code supporting the findings of this study are openly available at https://github.com/beaLando/floweringExp_linumBienne.git
