# Supplementary material for: Local climate and vernalization sensitivity predict the latitudinal patterns of flowering onset in the crop wild relative Linum bienne Mill
Source: Ann Bot. 2024 Mar 14;134(1):117–30. doi: 10.1093/aob/mcae040 (PMC11161566; doi:10.1093/aob/mcae040)
Supplement: mcae040_suppl_Supplementary_Tables [file mcae040_suppl_supplementary_tables.docx]

**Supplementary Table S1.** Surveyed *Linum bienne* populations and cultivars of *Linum usitatissimum*, with indication of the type of species (wild for *L. bienne*, type of cultivar as oilseed or fibre for *L. usitatissimum*). The table includes number of families collected in the field, collection date, country and locality, coordinates and elevation (meters above sea level), and cultivar provider. Column “Exp.” (Experiment) indicates which populations were used to describe flowering onset in the greenhouse (GH), vernalization response (V), and for genotyping (GT). For *L. bienne*, the seeds collected in the wild (F*_0_*) were directly used in experiments or propagated in the greenhouse to generate the F*_1_* used in the vernalization experiment. Populations were represented by different individual plants (families). For *L. usitatissimum,* all seeds represent a unique cultivar, and no distinction is done between individuals or families. Seeds provided by breeding companies and institutes were first propagated in the greenhouse for one generation before being used in the vernalization experiment. For *L. bienne*, coordinates represent the exact population's location; for *L. usitatissimum*, coordinates represent the centroid of the country of origin and no altitude is available.

| Species | Type | Population or Cultivar | Families Collected | Collected On | Country | Place of Collection or Provider | Latitude | Longitude | Altitude | Exp. |
| --- | --- | --- | --- | --- | --- | --- | --- | --- | --- | --- |
| *L. bienne* | wild | 1 | 47 | 04/06/2016 | Spain | Llanos del Rabel trail | 36.80044556 | -5.39258337 | 624 | GH, V |
| *L. bienne* | wild | 2 | 32 | 05/06/2016 | Spain | Virgen de la Luz Santuary Facinas | 36.08091736 | -5.625527859 | 41 | GH, V |
| *L. bienne* | wild | 3 | 41 | 05/06/2016 | Spain | Guadalmesi | 36.03633499 | -5.555888653 | 186 | GH, V |
| *L. bienne* | wild | 4 | 34 | 05/06/2016 | Spain | El Nene, Facinas | 36.15083313 | -5.704944611 | 36 | GH, V |
| *L. bienne* | wild | 5 | 35 | 07/06/2016 | Spain | Puebla del Río-Aznalcazar, Sevilla | 37.25852966 | -6.097222328 | 12 | GH, V |
| *L. bienne* | wild | 6 | 29 | 08/06/2016 | Spain | Constantina-Cazalla de la Sierra, Sevilla (trail) | 37.93551254 | -5.711172104 | 529 | GH, V, GT |
| *L. bienne* | wild | 7 | 21 | 08/06/2016 | Spain | Cardeña-Villa del Río, Cortijo Tejoneras, Córdoba | 38.25336075 | -4.317389011 | 752 | GH, V |
| *L. bienne* | wild | 8 | 27 | 08/06/2016 | Spain | Road 432, Km 12 Road to El Pedroso | 37.70194626 | -5.832055569 | 128 | GH, V |
| *L. bienne* | wild | 9 | 36 | 09/06/2016 | Spain | N-433 before exit to Zufre-La Granada de Riotinto, Huelva | 37.80849838 | -6.428805351 | 446 | GH, V |
| *L. bienne* | wild | 10 | 28 | 09/06/2016 | Spain | Linares de la Sierra, Huelva | 37.8821106 | -6.617805481 | 519 | GH, V |
| *L. bienne* | wild | 11 | 38 | 14/06/2016 | Spain | La Aliseda, Finca La Inmmediata (Km 3), Jaen | 38.33105087 | -3.580855608 | 710 | GH, V, GT |
| *L. bienne* | wild | 12 | 51 | 17/06/2016 | Spain | Palau-Savereda | 42.31008148 | 3.151888847 | 105 | GH, V |
| *L. bienne* | wild | 13 | 35 | 19/06/2016 | Spain | Quincoces de Yuso-Relloso, Burgos | 43.0290184 | -3.240350008 | 741 | GH, V |
| *L. bienne* | wild | 14 | 38 | 20/06/2016 | Spain | Tartales de Cilla | 42.79404068 | -3.424744368 | 670 | GH, V |
| *L. bienne* | wild | 15 | 32 | 20/06/2016 | Spain | Cantabria-Carriazo.Galizano | 43.46278381 | -3.653308392 | 50 | GH, V |
| *L. bienne* | wild | 19 | 31 | 15/07/2016 | Spain | Universidad de Vigo | 42.17088699 | -8.683819771 | 483 | GH, V |
| *L. bienne* | wild | BH | 13 | 09/07/2017 | UK | Barry Head | 50.40043259 | -3.492599964 | 55 | GH |
| *L. bienne* | wild | Bro | 39 | 04/07/2016 | France | Brossay, Maine et Loire | 47.16703796 | -0.207049996 | 69.5 | GH |
| *L. bienne* | wild | Dor | NA | NA | UK | Dorset / Emorsgate Seeds | 50.6 | -2.01 | NA | V |
| *L. bienne* | wild | CGa1 | 21 | 06/06/2017 | Italy | Capo Gallo, Sicily | 38.21649933 | 13.32183361 | 53 | GH |
| *L. bienne* | wild | CR | 30 | 08/07/2017 | UK | Cheddar Reservoir | 51.27793503 | -2.795016766 | 15 | GH |
| *L. bienne* | wild | IOW1 | 26 | 24/09/2016 | UK | Bembridge, 1st stop, Isle of Wight | 50.69146729 | -1.095399976 | 1 | V |
| *L. bienne* | wild | IOW2 | 42 | 24/09/2016 | UK | Bembridge, 2nd stop, Isle of Wight | 50.68183517 | -1.07491672 | 9 | GH, V, GT |
| *L. bienne* | wild | Lil | 21 | 09/07/2017 | UK | Lilstock | 51.20166779 | -3.186933279 | 23 | GH |
| *L. bienne* | wild | LJLb1 | 16 | 15/06/2014 | Spain | Las Juntas, Jaen | 38.221069 | -2.45397 | 1325 | GH |
| *L. bienne* | wild | Lla | 35 | 21/07/2014 | Spain | Llanes, Asturias | 43.40737534 | -4.687527657 | 26 | GH, V, GT |
| *L. bienne* | wild | Man | 41 | 10/09/2016 | UK | Mansfield, Nottinghamshire | 53.13731003 | -1.143669486 | 124 | GH, V |
| *L. bienne* | wild | Mat | 25 | 29/06/2016 | France | Mathaux, Aube | 48.35696793 | 4.458630562 | 125.5 | GH, V |
| *L. bienne* | wild | Roc | 40 | 04/07/2016 | France | Domaine de Rochambeau, Maine et Loire | 47.38702011 | -0.525738895 | 47.5 | GH, V |
| *L. bienne* | wild | Saf | 30 | 05/07/2016 | Spain | Saffre, Loire Atlantique | 47.49610138 | -1.592538834 | 25 | GH, V |
| *L. bienne* | wild | Sut | 42 | 09/09/2016 | UK | Sutton Cum Lound, Nottinghamshire | 53.3529129 | -0.959269464 | 15 | GH, V, GT |
| *L. bienne* | wild | Tal | 40 | 06/07/2016 | France | Pointe du Talude, Morbihan | 47.6996994 | -3.454649925 | 14 | GH, V |
| *L. bienne* | wild | Tor | 18 | 11/07/2013 | Spain | Torrox Costa, Malaga | 36.73955917 | -3.926350117 | 24 | V |
| *L. bienne* | wild | Tym | 40 | 02/09/2016 | UK | Tyr Mawr Holiday Park, Denbighshire | 53.30307388 | -3.553280592 | 5 | GH, V |
| *L. bienne* | wild | Vil | 38 | 02/07/2016 | France | Villeneuve, Charente Maritime | 45.09392929 | -1.050338864 | 21 | GH, V, GT |
| *L. usitatissimum* | cultivated, fibre spring | Aramis | - | - | France | Terre de Lin | 46.227638 | 2.213749 | - | V |
| *L. usitatissimum subsp. elongatum* | cultivated, fibre | Ariane | - | - | France | IPK | 46.227638 | 2.213749 | - | V |
| *L. usitatissimum subsp. elongatum* | cultivated, fibre | Blenda 04C | - | - | Netherlands | IPK | 52.132633 | 5.291266 | - | V |
| *L. usitatissimum* | cultivated, fibre spring | Bolchoi | - | - | France | Terre de Lin | 46.227638 | 2.213749 | - | V |
| *L. usitatissimum* | cultivated, fibre spring | Eden | - | - | France | Terre de Lin | 46.227638 | 2.213749 | - | V |
| *L. usitatissimum subsp. caesium* | cultivated, oilseed | Gisa | - | - | Italy | IPK | 41.87194 | 12.56738 | - | V |
| *L. usitatissimum subsp. caesium* | cultivated, oilseed | Liral Crown | - | - | UK | IPK | 55.378051 | -3.435973 | - | V |
| *L. usitatissimum* | cultivated, oilseed | Marmalade | - | - | Canada | Flaxland | 56.130366 | -106.346771 | - | V |
| *L. usitatissimum subsp. elongatum* | cultivated, fibre | Monarch | - | - | UK | IPK | 55.378051 | -3.435973 | - | V |
| *L. usitatissimum* | cultivated, fibre winter | Olga | - | - | France | Terre de Lin | 46.227638 | 2.213749 | - | V |
| *L. usitatissimum* | cultivated, oilseed spring | Omegalin | - | - | France | Terre de Lin | 46.227638 | 2.213749 | - | V |
| *L. usitatissimum subsp. mediterraneum* | cultivated, oilseed | Primus | - | - | Italy | IPK | 41.87194 | 12.56738 | - | V |
| *L. usitatissimum subsp. mediterraneum* | cultivated, oilseed | Raba 0189 | - | - | Morocco | IPK | 31.791702 | -7.09262 | - | V |
| *L. usitatissimum* | cultivated, fibre | Suzanne | - | - | Netherlands | Flaxland | 52.132633 | 5.291266 | - | V |
| *L. usitatissimum subsp. caesium* | cultivated, oilseed | Tine Tammes Lila | - | - | Netherlands | IPK | 52.132633 | 5.291266 | - | V |
| *L. usitatissimum* | cultivated, oilseed winter | Volga | - | - | France | Terre de Lin | 46.227638 | 2.213749 | - | V |

**Supplementary Table S2**. Summary results of the days to flowering onset measured in *Linum bienne* populations in the greenhouse experiment (*F_0_* generation) and in the vernalization experiment (*F_1_* generation), and the vernalization sensitivity (Vern. Sensitivity) for *Linum bienne* populations and *Linum usitatissimum* cultivars. For the greenhouse experiment, the columns “Sown” and “Flowered” indicate the number of sown families and the number of families that flowered, and the column “Flowering Onset” indicates the population mean (m) and standard deviation (sd). For the vernalization experiment, data and sample size are represented with the distinction of the no vernalization (NV) and vernalization (V) treatment. The column “Sown” indicates the number of sown families, including the seed sample size used per treatment in parenthesis. The column “Flowered” includes the number of plants that flowered, followed by the number of families. The columns “Flowering Onset NV” and “Flowering onset V” include the mean (m) and standard deviation for the no vernalization and vernalization treatment, respectively. * Value not calculated because of n < 2 for at least one treatment.

| **Species** | **Population or Cultivar** | **Greenhouse experiment (*F_0_* plants)** | | | | **Vernalization experiment (*F_1_* Plants and Cultivars)** | | | | | |
| --- | --- | --- | --- | --- | --- | --- | --- | --- | --- | --- | --- |
|  |  | **Sown** | **Flowered** | **Flowering Onset** | **Sown (NV/V) Family (seeds)** | | **Flowered (NV/V)** | | **Flowering Onset NV** | **Flowering Onset V** | **Vernalization**  **Sensitivity (VRN)** |
|  |  |  |  |  |  |  | **No. plants** | **No. families** |  |  |  |
| *L. bienne* | 1 | 30 | 20 | m = 149, sd = 21 | 6/6 (55/60) | | 6/4 | 4/2 | m = 155, sd = 23 | m = 97, sd = 2 | 0.77 |
| *L. bienne* | 2 | 30 | 29 | m = 137, sd = 23 | 2/2 (20/20) | | 3/4 | 2/2 | m = 153, sd = 66 | m = 99, sd = 10 | 0.73 |
| *L. bienne* | 3 | 29 | 12 | m = 189, sd = 29 | 4/4 (40/40) | | 8/7 | 4/4 | m = 172, sd = 30 | m = 104, sd = 3 | 0.91 |
| *L. bienne* | 4 | 27 | 20 | m = 139, sd = 21 | 5/6 (50/65) | | 8/8 | 5/5 | m = 110, sd = 47 | m = 95, sd = 5 | 0.2 |
| *L. bienne* | 5 | 30 | 23 | m = 139, sd = 18 | 2/2 (20/25) | | 2/5 | 2/2 | m = 77, sd = 13 | m = 89, sd = 2 | -0.17 |
| *L. bienne* | 6 | 29 | 20 | m = 190, sd = 22 | 3/3 (30/35) | | 4/4 | 3/2 | m = 182, sd = 45 | m = 102, sd = 4 | 1.07 |
| *L. bienne* | 7 | 21 | 15 | m = 197, sd = 23 | 1/1 (10/10) | | 1/0 | 1/0 | m = 134, n = 1 | - | * |
| *L. bienne* | 8 | 24 | 18 | m = 176, sd = 31 | 4/4 (40/40) | | 3/3 | 2/2 | m = 197, sd = 25 | m = 93, sd = 5 | 1.4 |
| *L. bienne* | 9 | 30 | 25 | m = 129, sd = 18 | 3/3 (30/30) | | 4/2 | 3/2 | m = 97, sd = 30 | m = 91, sd = 1 | 0.09 |
| *L. bienne* | 10 | 28 | 26 | m = 175, sd = 29 | 5/4 (40/40) | | 7/6 | 4/4 | m = 122, sd = 23 | m = 88, sd = 5 | 0.45 |
| *L. bienne* | 11 | 30 | 16 | m = 179, sd = 36 | 1/1 (10/10) | | 1/1 | 1/1 | m = 171 , n = 1 | m = 92, n = 1 | * |
| *L. bienne* | 12 | 30 | 10 | m = 209, sd = 24 | 1/1 (10/10) | | 0/1 | 0/1 | - | m = 95, n = 1 | * |
| *L. bienne* | 13 | 30 | 4 | m = 185, sd = 61 | 3/4 (30/40) | | 1/4 | 1/3 | m = 204, n = 1 | m = 115, sd = 20 | * |
| *L. bienne* | 14 | 8 | 1 | m = 195, sd = NA | 2/2 (20/20) | | 1/3 | 1/2 | m = 223, n = 1 | m = 108, sd = 5 | * |
| *L. bienne* | 15 | 31 | 12 | m = 200, sd = 24 | 6/5 (55/50) | | 7/10 | 4/5 | m = 187, sd = 32 | m = 103, sd = 6 | 1.13 |
| *L. bienne* | 19 | 30 | 20 | m = 217, sd = 23 | 2/2 (25/20) | | 3/2 | 2/1 | m = 207, sd = 17 | m = 112, sd = 6 | 1.27 |
| *L. bienne* | BH | 12 | 3 | m = 228, sd = 7 | - | | - | | - | - | - |
| *L. bienne* | Bro | 29 | 6 | m = 194, sd = 38 | - | | - | | - | - | - |
| *L. bienne* | CGa1 | 21 | 21 | m = 137, sd = 21 | - | | - | | - | - | - |
| *L. bienne* | CR | 30 | 4 | m = 221, sd = 15 | - | | - | | - | - | - |
| *L. bienne* | IOW1 | - | - | - | 3/3 (30/30) | | 2/6 | 2/3 | m = 303, sd = 25 | m = 105, sd = 7 | 2.65 |
| *L. bienne* | DOR | - | - | - | 3/3 (35/30) | | 0/6 | 0/2 | - | m = 110, sd = 5 | * |
| *L. bienne* | IOW2 | 30 | 11 | m = 195, sd = 49 | 4/4 (40/40) | | 5/8 | ¾ | m = 103, sd = 68 | m = 107, sd = 10 | -0.06 |
| *L. bienne* | Lil | 21 | 4 | m = 201, sd = 27 | - | | - | | - | - | - |
| *L. bienne* | LJLb1 | 16 | 9 | m = 209, sd = 30 | - | | - | | - | - | - |
| *L. bienne* | Lla | 30 | 16 | m = 202, sd = 30 | 9/8 (80/80) | | 7/12 | 6/7 | m = 248, sd = 38 | m = 113, sd = 12 | 1.8 |
| *L. bienne* | Man | 30 | 8 | m = 213, sd = 39 | 4/4 (45/35) | | 4/8 | ¾ | m = 241, sd = 40 | m = 106, sd = 3 | 1.8 |
| *L. bienne* | Mat | 25 | 22 | m = 208, sd = 21 | 5/5 (50/50) | | 10/10 | 5/5 | m = 213, sd = 20 | m = 108, sd = 9 | 1.41 |
| *L. bienne* | Roc | 29 | 16 | m = 213, sd = 29 | 1/1 (10/10) | | 2/2 | 1/1 | m = 237, sd = 33 | m = 103, sd = 4 | 1.8 |
| *L. bienne* | Saf | 27 | 7 | m = 186, sd = 37 | 4/4 (40/40) | | 4/8 | ¾ | m = 264, sd = 53 | m = 108, sd = 6 | 2.1 |
| *L. bienne* | Sut | 29 | 8 | m = 208, sd = 24 | 1/1 (5/5) | | 0/2 | 0/1 | - | m = 113, sd = 0.7 | * |
| *L. bienne* | Tal | 28 | 9 | m = 208, sd = 30 | 4/4 (40/40) | | 2/7 | ¼ | m = 120, sd = 53 | m = 116, sd = 15 | 0.05 |
| *L. bienne* | Tor | - | - | - | 1/1 (10/10) | | 2/2 | 1/1 | m = 76, sd = 6 | m = 88, sd = 2 | -0.15 |
| *L. bienne* | Tym | 30 | 8 | m = 224, sd = 10 | 4/4 (35/ 40) | | 2/8 | 2/4 | m = 190, sd = 28 | m = 112, sd = 12 | 1.04 |
| *L. bienne* | Vil | 30 | 21 | m = 205, sd = 31 | 4/4 (40/40) | | 4/8 | ¾ | m = 234, sd = 47 | m = 107, sd = 4 | 1.71 |
| *L. usitatissimum* | Aramis | - | - | - | (10/10) | | 2/2 | 1/1 | m = 85, sd = 1 | m = 97, sd = 0 | 0.47 |
| *L. usitatissimum* | Ariane | - | - | - | (20/20) | | 3/4 | 1/1 | m = 66, sd = 9 | m = 92, sd = 4 | 1.03 |
| *L. usitatissimum* | Blenda 04C | - | - | - | (20/20) | | 4/4 | 1/1 | m = 56, sd = 4 | m = 101, sd = 5 | 1.75 |
| *L. usitatissimum* | Bolchoi | - | - | - | (10/10) | | 2/2 | 1/1 | m = 70, sd = 7 | m = 95, sd = 4 | 0.96 |
| *L. usitatissimum* | Eden | - | - | - | (10/10) | | 3/2 | 1/1 | m = 73, sd = 2 | m = 102, sd = 4 | 1.11 |
| *L. usitatissimum* | Gisa | - | - | - | (20/20) | | 4/4 | 1/1 | m = 78, sd = 8 | m = 103, sd = 7 | 0.97 |
| *L. usitatissimum* | Liral Crown | - | - | - | (20/10) | | 3/2 | 1/1 | m = 60, sd = 4 | m = 87, sd = 6 | 1.05 |
| *L. usitatissimum* | Marmalade | - | - | - | (10/10) | | 2/2 | 1/1 | m = 66, sd = 6 | m = 96, sd = 1 | 1.16 |
| *L. usitatissimum* | Monarch | - | - | - | (20/25) | | 4/4 | 1/1 | m = 58, sd = 6 | m = 96, sd = 5 | 1.47 |
| *L. usitatissimum* | Olga | - | - | - | (10/10) | | 2/2 | 1/1 | m = 111, sd = 19 | m = 100, sd = 4 | -0.43 |
| *L. usitatissimum* | Omegalin | - | - | - | (20/10) | | 4/2 | 1/1 | m = 58, sd = 4 | m = 86, sd = 1 | 1.12 |
| *L. usitatissimum* | Primus | - | - | - | (20/20) | | 4/4 | 1/1 | m = 90, sd = 34 | m = 104, sd = 15 | 0.53 |
| *L. usitatissimum* | Raba 0189 | - | - | - | (20/20) | | 4/4 | 1/1 | m = 75, sd = 34 | m = 98, sd = 4 | 0.92 |
| *L. usitatissimum* | Suzanne | - | - | - | (20/10) | | 4/2 | 1/1 | m = 62, sd = 18 | m = 99, sd = 0 | 1.46 |
| *L. usitatissimum* | Tine Tammes Lila | - | - | - | (20/20) | | 4/4 | 1/1 | m = 71, sd = 3 | m = 101, sd = 6 | 1.2 |
| *L. usitatissimum* | Volga | - | - | - | (10/10) | | 2/2 | 1/1 | m = 68, sd = 6 | m = 99, sd = 1 | 1.22 |

**Supplementary Table S3.** Population scores of the PC axis PC1 to PC3 retrieved from the principal component analysis using climatic variables obtained from WorldClim (see Material and Methods for variable details).

| Population  code | PC1 | PC2 | PC3 |
| --- | --- | --- | --- |
| 1 | -2.291467828 | -1.290486714 | -0.489630256 |
| 10 | -2.852556155 | -1.925370509 | 0.037314959 |
| 11 | -2.07186556 | -3.918679217 | 1.071100071 |
| 12 | -0.733578784 | 1.851462156 | 2.772616161 |
| 13 | 3.173802349 | -2.374054198 | -2.199141064 |
| 14 | 2.740272062 | -3.436562987 | -1.511602566 |
| 15 | -0.335266075 | 1.400913892 | -3.2729147 |
| 19 | 1.03050902 | 2.46611844 | -6.189634746 |
| 2 | -6.63777471 | 2.979480244 | -0.855788691 |
| 3 | -5.68853179 | 2.867174217 | -0.252056176 |
| 4 | -6.767529443 | 2.245956561 | -1.232452184 |
| 5 | -6.592747951 | -0.967980449 | 0.352557295 |
| 6 | -3.031496968 | -2.160163803 | 0.226964613 |
| 7 | -1.658589248 | -3.683390778 | 0.360709324 |
| 8 | -5.919001921 | -1.524702281 | 0.516137584 |
| 9 | -3.477539346 | -1.987814503 | 0.156886035 |
| BH | 4.50509557 | 3.708187106 | 1.540203127 |
| Bro | 2.586605006 | -0.895439633 | 0.195889907 |
| CGa1 | -6.198109461 | 2.646379021 | 2.057210102 |
| CR | 4.559435062 | 0.019817495 | -0.510024285 |
| IOW2 | 4.58438405 | 2.13301505 | 1.207342576 |
| Lil | 4.925002897 | 1.860968404 | 0.188505061 |
| LJLb1 | -0.596877675 | -4.146628428 | 1.436046952 |
| Lla | -0.410226972 | 0.020807429 | -1.560793833 |
| Man | 6.398702396 | -0.436962799 | 0.906954086 |
| Mat | 3.827236743 | -2.106673306 | -0.248804619 |
| Roc | 2.359780451 | -0.809307141 | 0.130227823 |
| Saf | 2.142652953 | -0.716130927 | -0.454861922 |
| Sut | 5.763015137 | -0.81807769 | 1.491577329 |
| Tal | 2.353862723 | 3.506399837 | 0.83780612 |
| Tym | 5.710194144 | 1.455560392 | 1.154050848 |
| Vil | 0.853559975 | 0.246823142 | -1.043774482 |
| IOW1 | 4.598915497 | 2.156614411 | 1.233527246 |
| Tor | -6.849866147 | 1.632747567 | 1.947852306 |

**Supplementary Table S4.** Pearson's correlations between climatic PC1, PC2, PC3, and flowering onset of *Linum bienne* populations measured in the greenhouse (Flowering Onset *F_0_*), and in the vernalization experiment using the *F_1_* (Flowering Onset *F_1_* No-Vern. and Vern. for the no vernalization and vernalization treatments, respectively, and Vernalization Sensitivity). Pearson's correlation coefficients between each pair of variables (r) are reported in the lower triangle and in bold when statistically significant (p < 0.05). Sample sizes (n) on which correlation coefficients between each pair of variables were calculated are reported in the upper triangle. Sample sizes vary between pairs of variables because not all populations were used across all experiments.

| *Variables* | Latitude | climatic PC1 | climatic PC2 | climatic PC3 | Flowering Onset F0 | Flowering Onset F1 No-Vern | Flowering Onset F1 Vern. | Vernalization Sensitivity |
| --- | --- | --- | --- | --- | --- | --- | --- | --- |
| Latitude |  | 34 | 34 | 34 | 32 | 22 | 22 | 22 |
| climatic PC1 | **0.932** |  | 34 | 34 | 32 | 22 | 22 | 22 |
| climatic PC2 | 0.223 | 0.000 |  | 34 | 32 | 22 | 22 | 22 |
| climatic PC3 | 0.126 | 0.000 | 0.000 |  | 32 | 22 | 22 | 22 |
| Flowering Onset F0 | **0.708** | **0.781** | 0.056 | -0.017 |  | 20 | 20 | 20 |
| Flowering Onset F1  No-Vern. | **0.541** | **0.573** | -0.090 | -0.197 | **0.642** |  | 22 | 22 |
| Flowering Onset F1 Vern. | **0.700** | **0.730** | 0.372 | -0.256 | **0.797** | **0.574** |  | 22 |
| Vernalization Sensitivity | **0.481** | **0.511** | -0.150 | -0.175 | **0.564** | **0.993** | **0.474** |  |

**Supplementary Table S5.** Estimate values of the fixed terms, interactions terms and random factors derived from the linear mixed effect model on flowering onset of *Linum bienne* in the vernalization experiment.

| Predictors | Estimates | CI | P value | df |
| --- | --- | --- | --- | --- |
| (Intercept) | 144.17 | 121.93 – 166.42 | <0.001 | 118.92 |
| Number of basal branches | 1.33 | 0.62 – 2.05 | <0.001 | 185.56 |
| Pop [10] | -26.69 | -54.96 – 1.57 | 0.064 | 98.09 |
| Pop [15] | 31.70 | 3.31 – 60.10 | 0.029 | 107.72 |
| Pop [19] | 37.76 | 1.42 – 74.11 | 0.042 | 111.31 |
| Pop [2] | -2.06 | -38.14 – 34.02 | 0.910 | 112.39 |
| Pop [3] | 16.11 | -11.81 – 44.02 | 0.255 | 97.75 |
| Pop [4] | -43.30 | -70.79 – -15.82 | 0.002 | 110.37 |
| Pop [5] | -75.03 | -115.22 – -34.85 | <0.001 | 149.17 |
| Pop [6] | 19.23 | -15.24 – 53.69 | 0.272 | 139.52 |
| Pop [8] | 33.25 | -2.59 – 69.08 | 0.069 | 110.33 |
| Pop [9] | -55.39 | -88.14 – -22.63 | 0.001 | 127.09 |
| Pop [IOW1] | 133.85 | 93.11 – 174.59 | <0.001 | 162.60 |
| Pop [IOW2] | -51.74 | -82.48 – -21.01 | 0.001 | 111.21 |
| Pop [Lla] | 81.81 | 53.08 – 110.54 | <0.001 | 132.91 |
| Pop [Man] | 76.70 | 43.69 – 109.70 | <0.001 | 128.79 |
| Pop [Mat] | 47.27 | 20.81 – 73.74 | 0.001 | 101.13 |
| Pop [Roc] | 60.77 | 17.32 – 104.23 | 0.007 | 93.27 |
| Pop [Saf] | 70.80 | 34.48 – 107.12 | <0.001 | 156.02 |
| Pop [Tal] | -33.63 | -76.29 – 9.04 | 0.121 | 89.46 |
| Pop [Tor] | -76.94 | -118.00 – -35.88 | <0.001 | 79.92 |
| Pop [Tym] | 22.01 | -17.17 – 61.18 | 0.269 | 158.54 |
| Pop [Vil] | 78.80 | 46.15 – 111.46 | <0.001 | 130.35 |
| treatment [Vernalization] | -52.84 | -83.33 – -22.34 | 0.001 | 177.98 |
| Pop [10] × treatment[Vernalization] | 17.11 | -21.46 – 55.69 | 0.382 | 159.53 |
| Pop [15] × treatment[Vernalization] | -25.62 | -62.44 – 11.21 | 0.171 | 166.31 |
| Pop [19] × treatment[Vernalization] | -28.45 | -81.39 – 24.49 | 0.290 | 182.72 |
| Pop [2] × treatment[Vernalization] | 2.60 | -42.02 – 47.23 | 0.908 | 151.39 |
| Pop [3] × treatment[Vernalization] | -7.39 | -45.43 – 30.65 | 0.702 | 171.63 |
| Pop [4] × treatment[Vernalization] | 45.36 | 8.12 – 82.61 | 0.017 | 171.32 |
| Pop [5] × treatment[Vernalization] | 69.98 | 22.68 – 117.27 | 0.004 | 148.72 |
| Pop [6] × treatment[Vernalization] | -17.99 | -63.23 – 27.25 | 0.433 | 162.40 |
| Pop [8] × treatment[Vernalization] | -34.74 | -83.29 – 13.82 | 0.160 | 182.00 |
| Pop [9] × treatment[Vernalization] | 51.01 | 1.36 – 100.65 | 0.044 | 175.69 |
| Pop [IOW1] × treatment[Vernalization] | -124.94 | -172.98 – -76.89 | <0.001 | 160.49 |
| Pop [IOW2] × treatment[Vernalization] | 57.28 | 18.09 – 96.47 | 0.004 | 163.48 |
| Pop [Lla] × treatment[Vernalization] | -65.76 | -102.97 – -28.55 | 0.001 | 173.85 |
| Pop [Man] × treatment[Vernalization] | -67.63 | -108.27 – -26.99 | 0.001 | 167.55 |
| Pop [Mat] × treatment[Vernalization] | -35.89 | -71.67 – -0.11 | 0.049 | 166.40 |
| Pop [Roc] × treatment[Vernalization] | -55.52 | -109.03 – -2.00 | 0.042 | 147.31 |
| Pop [Saf] × treatment[Vernalization] | -59.24 | -102.64 – -15.85 | 0.008 | 167.50 |
| Pop [Tal] × treatment[Vernalization] | 52.02 | 0.94 – 103.10 | 0.046 | 129.53 |
| Pop [Tor] × treatment[Vernalization] | 68.72 | 16.94 – 120.49 | 0.010 | 133.74 |
| Pop [Tym] × treatment[Vernalization] | -6.49 | -52.37 – 39.39 | 0.780 | 160.94 |
| Pop [Vil] × treatment[Vernalization] | -67.90 | -108.46 – -27.34 | 0.001 | 165.41 |
| Random Effects |  |  |  |  |
| σ2 | 431.87 |  |  | 431.87 |
| τ00 Population:Family | 100.02 |  |  | 100.02 |
| τ00treatment:SowDate:block | 87.24 |  |  | 87.24 |
| ICC | 0.30 |  |  | 0.30 |
| N Population | 22 |  |  | 22 |
| N Family | 85 |  |  | 85 |
| N treatment | 2 |  |  | 2 |
| N SowDate | 4 |  |  | 4 |
| N block | 20 |  |  | 20 |
| Observations | 232 |  |  | 232 |
| Marginal R2 / Conditional R2 | 0.812 / 0.869 |  |  | 0.812 / 0.869 |

**Supplementary Table S6.** Estimate values of the fixed terms, interactions terms and random factors derived from the linear mixed effect model on flowering onset of *Linum usitatissimum* in the vernalization experiment.

| Predictors | Estimates | CI | P value | Df |
| --- | --- | --- | --- | --- |
| (Intercept) | 75.10 | 60.30 – 89.89 | <0.001 | 62.37 |
| Number of basal branches | 5.47 | 3.62 – 7.32 | <0.001 | 63.64 |
| Pop [Ari] | -26.94 | -45.58 – -8.29 | 0.005 | 61.69 |
| Pop [Ble] | -27.39 | -44.98 – -9.80 | 0.003 | 61.22 |
| Pop [Bol] | -12.37 | -32.69 – 7.95 | 0.228 | 58.28 |
| Pop [Ede] | -8.14 | -26.77 – 10.49 | 0.386 | 63.06 |
| Pop [Gis] | -12.98 | -30.13 – 4.18 | 0.135 | 53.16 |
| Pop [Lir] | -27.89 | -45.87 – -9.91 | 0.003 | 52.60 |
| Pop [Mar] | -22.72 | -42.22 – -3.23 | 0.023 | 53.66 |
| Pop [Mon] | -25.25 | -42.81 – -7.68 | 0.006 | 60.62 |
| Pop [Olg] | 31.84 | 12.27 – 51.42 | 0.002 | 49.74 |
| Pop [Ome] | -23.53 | -41.10 – -5.96 | 0.010 | 59.95 |
| Pop [Pri] | -0.67 | -18.35 – 17.01 | 0.940 | 59.91 |
| Pop [Rab] | -21.97 | -39.38 – -4.56 | 0.014 | 55.92 |
| Pop [Suz] | -24.49 | -41.60 – -7.38 | 0.006 | 53.91 |
| Pop [Tin] | -13.30 | -30.88 – 4.28 | 0.135 | 60.31 |
| Pop [Vol] | -19.71 | -39.96 – 0.54 | 0.056 | 63.01 |
| treatment [Vernalization] | 15.04 | -5.20 – 35.28 | 0.143 | 60.64 |
| Pop [Ari] × treatment[Vernalization] | 18.85 | -6.07 – 43.77 | 0.135 | 55.65 |
| Pop [Ble] × treatment[Vernalization] | 30.46 | 5.95 – 54.98 | 0.016 | 58.09 |
| Pop [Bol] × treatment[Vernalization] | 10.93 | -17.20 – 39.05 | 0.439 | 53.87 |
| Pop [Ede] × treatment[Vernalization] | 13.69 | -13.76 – 41.13 | 0.323 | 62.79 |
| Pop [Gis] × treatment[Vernalization] | 10.59 | -13.89 – 35.07 | 0.390 | 57.58 |
| Pop [Lir] × treatment[Vernalization] | 14.71 | -12.34 – 41.75 | 0.281 | 56.67 |
| Pop [Mar] × treatment[Vernalization] | 21.67 | -6.44 – 49.78 | 0.128 | 58.70 |
| Pop [Mon] × treatment[Vernalization] | 23.73 | -1.08 – 48.54 | 0.061 | 60.35 |
| Pop [Olg] × treatment[Vernalization] | -26.92 | -55.04 – 1.19 | 0.060 | 56.14 |
| Pop [Ome] × treatment[Vernalization] | 13.12 | -13.64 – 39.88 | 0.331 | 61.57 |
| Pop [Pri] × treatment[Vernalization] | -9.49 | -34.16 – 15.17 | 0.444 | 56.22 |
| Pop [Rab] × treatment[Vernalization] | 18.68 | -5.26 – 42.62 | 0.124 | 51.74 |
| Pop [Suz] × treatment[Vernalization] | 27.93 | 1.41 – 54.46 | 0.039 | 58.09 |
| Pop [Tin] × treatment[Vernalization] | 14.48 | -9.79 – 38.75 | 0.237 | 53.40 |
| Pop [Vol] × treatment[Vernalization] | 20.95 | -7.70 – 49.60 | 0.149 | 62.19 |
| Random Effects |  |  |  |  |
| σ2 | 85.93 |  |  | 85.93 |
| τ00treatment:SowDate:block | 14.70 |  |  | 14.70 |
| ICC | 0.15 |  |  | 0.15 |
| N treatment | 2 |  |  | 2 |
| N SowDate | 4 |  |  | 4 |
| N block | 18 |  |  | 18 |
| Observations | 97 |  |  | 97 |
| Marginal R2 / Conditional R2 | 0.764 / 0.798 |  |  | 0.764 / 0.798 |

Supplementary Table S7. Results including the mean proportional membership of individuals across STRUCTURE runs obtained with CLUMPAK (option "Compare") for K = 2, when STRUCTURE was run considering microsatellite markers as dominant or co-dominant. For each individual within a population (rows), the mean proportional membership is reported for both clusters under each scenario (dominant or co-dominant markers). Notice that the cluster where membership was higher than 0.5 (blue highlights) always coincides across scenarios.

| **Population** | **Individual** | **K1_dominant** | **K2_dominant** | **K1_codominant** | **K2_codominant** |
| --- | --- | --- | --- | --- | --- |
| 11 | 1 | 0,004 | 0,996 | 0.0025 | 0.9975 |
| 11 | 10 | 0.0048 | 0.9952 | 0.0055 | 0.9945 |
| 11 | 11 | 0.002 | 0.998 | 0.0019 | 0.9981 |
| 11 | 12 | 0.002 | 0.998 | 0.0012 | 0.9988 |
| 11 | 13 | 0.002 | 0.998 | 0.0014 | 0.9986 |
| 11 | 14 | 0.002 | 0.998 | 0.0016 | 0.9984 |
| 11 | 15 | 0.002 | 0.998 | 0.0016 | 0.9984 |
| 11 | 16 | 0.002 | 0.998 | 0.0011 | 0.9989 |
| 11 | 17 | 0.002 | 0.998 | 0.0015 | 0.9985 |
| 11 | 19 | 0.002 | 0.998 | 0.0011 | 0.9989 |
| 11 | 20 | 0.002 | 0.998 | 0.0019 | 0.9981 |
| 11 | 21 | 0.0026 | 0.9974 | 0.0029 | 0.9971 |
| 11 | 22 | 0.002 | 0.998 | 0.001 | 0.999 |
| 11 | 23 | 0.002 | 0.998 | 0.0014 | 0.9986 |
| 11 | 25 | 0.048 | 0.952 | 0.0134 | 0.9866 |
| 11 | 26 | 0.004 | 0.996 | 0.0026 | 0.9974 |
| 11 | 31 | 0.005 | 0.995 | 0.0035 | 0.9965 |
| 11 | 32 | 0.0049 | 0.9951 | 0.0065 | 0.9935 |
| 11 | 5 | 0.002 | 0.998 | 0.0016 | 0.9984 |
| 11 | 6 | 0.002 | 0.998 | 0.0015 | 0.9985 |
| 11 | 7 | 0.002 | 0.998 | 0.0015 | 0.9985 |
| 11 | 8 | 0.002 | 0.998 | 0.0011 | 0.9989 |
| 11 | 9 | 0.002 | 0.998 | 0.0019 | 0.9981 |
| 6 | 1 | 0.004 | 0.996 | 0.0023 | 0.9977 |
| 6 | 10 | 0.002 | 0.998 | 0.0011 | 0.9989 |
| 6 | 12 | 0.002 | 0.998 | 0.0014 | 0.9986 |
| 6 | 13 | 0.0541 | 0.9459 | 0.1715 | 0.8285 |
| 6 | 14 | 0.003 | 0.997 | 0.0019 | 0.9981 |
| 6 | 15 | 0.002 | 0.998 | 0.0012 | 0.9988 |
| 6 | 17 | 0.003 | 0.997 | 0.0018 | 0.9982 |
| 6 | 19 | 0.004 | 0.996 | 0.002 | 0.998 |
| 6 | 2 | 0.002 | 0.998 | 0.0013 | 0.9987 |
| 6 | 21 | 0.0043 | 0.9957 | 0.0049 | 0.9951 |
| 6 | 22 | 0.0037 | 0.9963 | 0.002 | 0.998 |
| 6 | 23 | 0.004 | 0.996 | 0.0026 | 0.9974 |
| 6 | 25 | 0.002 | 0.998 | 0.0015 | 0.9985 |
| 6 | 26 | 0.002 | 0.998 | 0.0022 | 0.9978 |
| 6 | 27 | 0.005 | 0.995 | 0.0052 | 0.9948 |
| 6 | 28 | 0.0041 | 0.9959 | 0.0032 | 0.9968 |
| 6 | 29 | 0.003 | 0.997 | 0.0017 | 0.9983 |
| 6 | 30 | 0.047 | 0.953 | 0.0185 | 0.9815 |
| 6 | 4 | 0.002 | 0.998 | 0.0019 | 0.9981 |
| 6 | 6 | 0.007 | 0.993 | 0.0026 | 0.9974 |
| 6 | 7 | 0.002 | 0.998 | 0.001 | 0.999 |
| 6 | 8 | 0.0368 | 0.9632 | 0.0098 | 0.9902 |
| 6 | 9 | 0.002 | 0.998 | 0.0014 | 0.9986 |
| 18 | 1 | 0.998 | 0.002 | 0.9975 | 0.0025 |
| 18 | 10 | 0.998 | 0.002 | 0.9981 | 0.0019 |
| 18 | 11 | 0.998 | 0.002 | 0.9972 | 0.0028 |
| 18 | 12 | 0.9041 | 0.0959 | 0.9825 | 0.0175 |
| 18 | 13 | 0.998 | 0.002 | 0.9975 | 0.0025 |
| 18 | 14 | 0.998 | 0.002 | 0.9964 | 0.0036 |
| 18 | 15 | 0.998 | 0.002 | 0.9991 | 0.0009 |
| 18 | 16 | 0.998 | 0.002 | 0.9983 | 0.0017 |
| 18 | 17 | 0.998 | 0.002 | 0.9974 | 0.0026 |
| 18 | 18 | 0.994 | 0.006 | 0.9971 | 0.0029 |
| 18 | 19 | 0.998 | 0.002 | 0.9981 | 0.0019 |
| 18 | 2 | 0.9172 | 0.0828 | 0.9838 | 0.0162 |
| 18 | 20 | 0.998 | 0.002 | 0.9976 | 0.0024 |
| 18 | 22 | 0.998 | 0.002 | 0.9973 | 0.0027 |
| 18 | 23 | 0.998 | 0.002 | 0.9975 | 0.0025 |
| 18 | 25 | 0.998 | 0.002 | 0.9981 | 0.0019 |
| 18 | 27 | 0.998 | 0.002 | 0.998 | 0.002 |
| 18 | 28 | 0.998 | 0.002 | 0.9979 | 0.0021 |
| 18 | 29 | 0.998 | 0.002 | 0.9988 | 0.0012 |
| 18 | 30 | 0.998 | 0.002 | 0.9968 | 0.0032 |
| 18 | 4 | 0.997 | 0.003 | 0.9978 | 0.0022 |
| 18 | 6 | 0.997 | 0.003 | 0.9971 | 0.0029 |
| 18 | 8 | 0.997 | 0.003 | 0.9975 | 0.0025 |
| 18 | 9 | 0.9988 | 0.0012 | 0.9986 | 0.0014 |
| 31 | 1 | 0.996 | 0.004 | 0.996 | 0.004 |
| 31 | 10 | 0.9648 | 0.0352 | 0.9949 | 0.0051 |
| 31 | 11 | 0.996 | 0.004 | 0.9972 | 0.0028 |
| 31 | 12 | 0.9673 | 0.0327 | 0.997 | 0.003 |
| 31 | 13 | 0.4083 | 0.5917 | 0.3051 | 0.6949 |
| 31 | 14 | 0.0792 | 0.9208 | 0.0434 | 0.9566 |
| 31 | 16 | 0.996 | 0.004 | 0.998 | 0.002 |
| 31 | 17 | 0.0157 | 0.9843 | 0.004 | 0.996 |
| 31 | 18 | 0.7992 | 0.2008 | 0.8715 | 0.1285 |
| 31 | 19 | 0.99 | 0.01 | 0.9985 | 0.0015 |
| 31 | 2 | 0.9658 | 0.0342 | 0.9915 | 0.0085 |
| 31 | 22 | 0.993 | 0.007 | 0.9982 | 0.0018 |
| 31 | 28 | 0.996 | 0.004 | 0.9986 | 0.0014 |
| 31 | 3 | 0.987 | 0.013 | 0.9954 | 0.0046 |
| 31 | 30 | 0.997 | 0.003 | 0.9988 | 0.0012 |
| 31 | 32 | 0.9871 | 0.0129 | 0.9956 | 0.0044 |
| 31 | 33 | 0.987 | 0.013 | 0.9963 | 0.0037 |
| 31 | 34 | 0.994 | 0.006 | 0.9986 | 0.0014 |
| 31 | 35 | 0.9829 | 0.0171 | 0.9929 | 0.0071 |
| 31 | 5 | 0.987 | 0.013 | 0.9979 | 0.0021 |
| 31 | 8 | 0.279 | 0.721 | 0.3318 | 0.6682 |
| 31 | 9 | 0.7697 | 0.2303 | 0.8574 | 0.1426 |
| 31 | 37 | 0.9218 | 0.0782 | 0.997 | 0.003 |
| 31 | 38 | 0.996 | 0.004 | 0.9975 | 0.0025 |
| 31 | 39 | 0.993 | 0.007 | 0.9981 | 0.0019 |
| 31 | 40 | 0.9519 | 0.0481 | 0.9974 | 0.0026 |
| 39 | 10 | 0.998 | 0.002 | 0.9976 | 0.0024 |
| 39 | 11 | 0.998 | 0.002 | 0.998 | 0.002 |
| 39 | 13 | 0.998 | 0.002 | 0.9991 | 0.0009 |
| 39 | 14 | 0.998 | 0.002 | 0.9972 | 0.0028 |
| 39 | 15 | 0.998 | 0.002 | 0.9982 | 0.0018 |
| 39 | 16 | 0.998 | 0.002 | 0.9976 | 0.0024 |
| 39 | 18 | 0.998 | 0.002 | 0.9975 | 0.0025 |
| 39 | 2 | 0.998 | 0.002 | 0.9975 | 0.0025 |
| 39 | 20 | 0.998 | 0.002 | 0.9986 | 0.0014 |
| 39 | 21 | 0.998 | 0.002 | 0.9985 | 0.0015 |
| 39 | 24 | 0.998 | 0.002 | 0.9989 | 0.0011 |
| 39 | 26 | 0.004 | 0.996 | 0.0012 | 0.9988 |
| 39 | 27 | 0.998 | 0.002 | 0.9977 | 0.0023 |
| 39 | 28 | 0.998 | 0.002 | 0.9978 | 0.0022 |
| 39 | 29 | 0.998 | 0.002 | 0.9991 | 0.0009 |
| 39 | 3 | 0.119 | 0.881 | 0.1641 | 0.8359 |
| 39 | 30 | 0.998 | 0.002 | 0.9973 | 0.0027 |
| 39 | 32 | 0.998 | 0.002 | 0.9986 | 0.0014 |
| 39 | 33 | 0.0055 | 0.9945 | 0.002 | 0.998 |
| 39 | 34 | 0.989 | 0.011 | 0.9989 | 0.0011 |
| 39 | 36 | 0.998 | 0.002 | 0.997 | 0.003 |
| 39 | 37 | 0.996 | 0.004 | 0.998 | 0.002 |
| 39 | 38 | 0.998 | 0.002 | 0.998 | 0.002 |
| 39 | 39 | 0.998 | 0.002 | 0.9979 | 0.0021 |
| 39 | 4 | 0.998 | 0.002 | 0.9977 | 0.0023 |
| 39 | 40 | 0.998 | 0.002 | 0.9967 | 0.0033 |
| 39 | 41 | 0.998 | 0.002 | 0.9985 | 0.0015 |
| 39 | 42 | 0.998 | 0.002 | 0.9976 | 0.0024 |
| 39 | 6 | 0.998 | 0.002 | 0.9986 | 0.0014 |
| 39 | 9 | 0.998 | 0.002 | 0.9986 | 0.0014 |
| 43 | 1 | 0.998 | 0.002 | 0.9987 | 0.0013 |
| 43 | 10 | 0.9983 | 0.0017 | 0.9981 | 0.0019 |
| 43 | 11 | 0.9981 | 0.0019 | 0.9993 | 0.0007 |
| 43 | 12 | 0.9981 | 0.0019 | 0.9981 | 0.0019 |
| 43 | 16 | 0.999 | 0.001 | 0.9981 | 0.0019 |
| 43 | 17 | 0.998 | 0.002 | 0.999 | 0.001 |
| 43 | 18 | 0.9985 | 0.0015 | 0.9987 | 0.0013 |
| 43 | 19 | 0.998 | 0.002 | 0.9983 | 0.0017 |
| 43 | 2 | 0.998 | 0.002 | 0.9987 | 0.0013 |
| 43 | 21 | 0.696 | 0.304 | 0.6814 | 0.3186 |
| 43 | 22 | 0.999 | 0.001 | 0.9987 | 0.0013 |
| 43 | 23 | 0.998 | 0.002 | 0.9984 | 0.0016 |
| 43 | 24 | 0.9981 | 0.0019 | 0.998 | 0.002 |
| 43 | 25 | 0.9982 | 0.0018 | 0.9982 | 0.0018 |
| 43 | 26 | 0.998 | 0.002 | 0.9988 | 0.0012 |
| 43 | 27 | 0.998 | 0.002 | 0.9981 | 0.0019 |
| 43 | 28 | 0.9982 | 0.0018 | 0.9977 | 0.0023 |
| 43 | 29 | 0.998 | 0.002 | 0.9985 | 0.0015 |
| 43 | 3 | 0.999 | 0.001 | 0.9988 | 0.0012 |
| 43 | 30 | 0.998 | 0.002 | 0.9979 | 0.0021 |
| 43 | 32 | 0.999 | 0.001 | 0.999 | 0.001 |
| 43 | 35 | 0.9981 | 0.0019 | 0.998 | 0.002 |
| 43 | 36 | 0.998 | 0.002 | 0.9977 | 0.0023 |
| 43 | 37 | 0.9983 | 0.0017 | 0.9979 | 0.0021 |
| 43 | 38 | 0.998 | 0.002 | 0.9986 | 0.0014 |
| 43 | 4 | 0.999 | 0.001 | 0.9989 | 0.0011 |
| 43 | 5 | 0.997 | 0.003 | 0.9968 | 0.0032 |
| 43 | 6 | 0.999 | 0.001 | 0.9992 | 0.0008 |
| 43 | 7 | 0.999 | 0.001 | 0.9989 | 0.0011 |
| 43 | 8 | 0.9981 | 0.0019 | 0,9986 | 0,0014 |
