# Supplementary material for: Local climate and vernalization sensitivity predict the latitudinal patterns of flowering onset in the crop wild relative Linum bienne Mill
Source: Ann Bot. 2024 Mar 14;134(1):117–30. doi: 10.1093/aob/mcae040 (PMC11161566; doi:10.1093/aob/mcae040)

**Supplementary Figure S1.** Results of the principal component analyses to summarise the local climate of the populations surveyed along the latitudinal gradient, using data on mean monthly averages over 30-year period (1970 - 2000) for precipitation (mm), solar radiation (kJ/day*m2), average temperature (°C), minimum and maximum temperature (°C), vapour pressure (kPa), and wind speed (m/s) were retrieved using the WorldClim database at 30 arcsec resolution (Fick and Hijmans 2017). The climatic data were averaged by season. Months were assigned to seasons as follows: winter (December-February, DEF), spring (March-May, MAM), summer (June-August, JJA), and autumn (September-November, SON). The plots A and B represent PC1 (Dim1) against PC2 (Dim2, A), and PC1 against PC3 (Dim 3, B), including de % variance explained by each axis, with the contribution of each variable. Figure C includes the % contribution of each variable to PC1 (black), PC2 (grey), and PC3 (white).

|   B  A |  |
| --- | --- |
|   C |  |

Supplementary figure 2. Evanno’s method plot to retrieve optimal number of clusters for *Linum bienne* based on STRUCTURE output. (A) Mean likelihood for each K value, (B) Rate of change of the likelihood distribution, (C) Absolute values of the second order rate of change of the likelihood distribution, (D) Number of possible clusters, Delta K = 2 indicates the maximum K value. An examination of all parameters shows that there is an agreement with the optimal number of clusters K being 2.


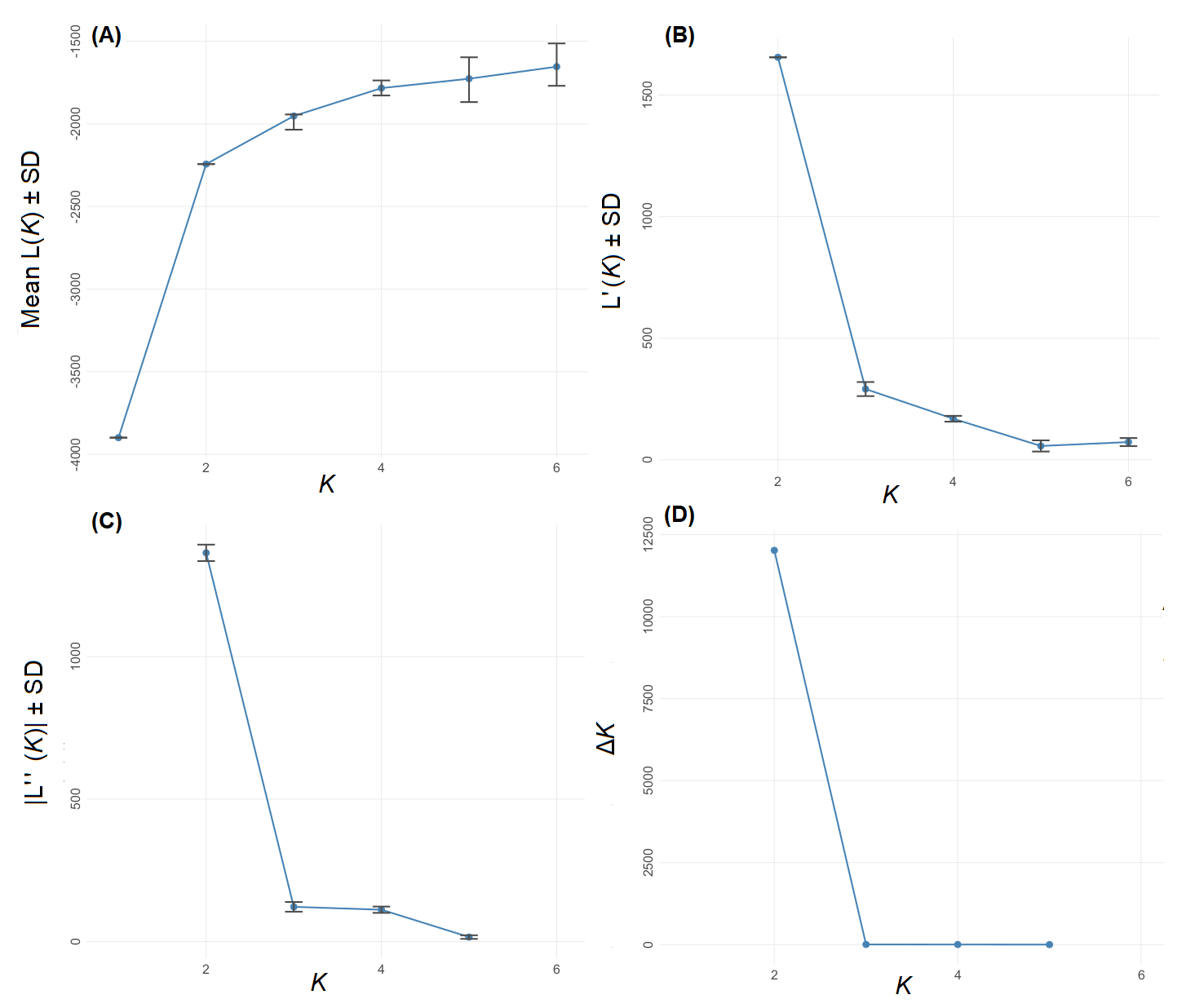

Supplement: mcae040_suppl_Supplementary_Figures [file mcae040_suppl_supplementary_figures.docx]
